# Flavonoids from *Machilus japonica* Stems and Their Inhibitory Effects on LDL Oxidation

**DOI:** 10.3390/ijms150916418

**Published:** 2014-09-16

**Authors:** Se-Jin Joo, Hee-Jung Park, Ji-Hae Park, Jin-Gyeong Cho, Ji-Hyun Kang, Tae-Sook Jeong, Hee Cheol Kang, Dae-Young Lee, Hack-Soo Kim, Sang-Yo Byun, Nam-In Baek

**Affiliations:** 1 R&D Center, Seoul Cosmetics Co., Ltd., Incheon 443-749, Korea; E-Mails: skincare@seoulcos.co.kr (S.-J.J.); hskim@seoulcos.co.kr (H.-S.K.); 2 Laboratory of Natural Products Chemistry, Graduate School of Oriental Medicinal Materials Biotechnology, Kyung Hee University, Yongin 446-701, Korea; E-Mails: bluedew011@hanmail.net (H.-J.P.); wlgo3411@hanmail.net (J.-H.P.); kukuku1555@khu.ac.kr (J.-G.J.); 3 National Research Laboratory of Lipid Metabolism & Atherosclerosis, KRIBB, Daejeon 305-806, Korea; E-Mails: bsloaucl@naver.com (J.-H.K.); tsjeong@kribb.re.kr (T.-S.J.); 4 Research & Development Center, GFC Co., Ltd., Suwon 443-813, Korea; E-Mail: cmkorea@unitel.co.kr; 5 Department of Herbal Crop Research, National Institute of Horticultural and Herbal Science, RDA, Eumseong 369-873, Korea; E-Mail: dylee0809@korea.kr; 6 Cosmetic Business R&D Service Center, Graduate School, Ajou University, Suwon 443-749, Korea; E-Mail: sybyun@ajou.ac.kr

**Keywords:** diphenyl picryl hydrazinyl, low-density lipoprotein-oxidation, flavonoid, *Machilus japonica*, NMR

## Abstract

Stems of *Machilus japonica* were extracted with 80% aqueous methanol (MeOH) and the concentrated extract was successively extracted with ethyl acetate (EtOAc), normal butanol (*n*-BuOH), and water. Six flavonoids were isolated from the EtOAc fraction: (+)-taxifolin, afzelin, (−)-epicatechin, 5,3'-di-*O*-methyl-(−)-epicatechin, 5,7,3'-tri-*O*-methyl-(−)-epicatechin, and 5,7-di-*O*-methyl-3',4'-methylenedioxyflavan-3-ol. The chemical structures were identified using spectroscopic data including NMR, mass spectrometry and infrared spectroscopy. This is the first report of isolation of these six compounds from *M. japonica*. The compounds were evaluated for their diphenyl picryl hydrazinyl scavenging activity and inhibitory effects on low-density lipoprotein oxidation. Compounds **1** and **3**–**6** exhibited DPPH antioxidant activity equivalent with that of ascorbic acid, with half maximal inhibitory concentration (IC_50_) values of 0.16, 0.21, 0.17, 0.15 and 0.07 mM, respectively. The activity of compound **1** was similar to the positive control butylated hydroxytoluene, which had an IC_50_ value of 1.9 µM, while compounds **3** and **5** showed little activity. Compounds **1**, **3**, and **5** exhibited LDL antioxidant activity with IC_50_ values of 2.8, 7.1, and 4.6 µM, respectively.

## 1. Introduction

The family Lauraceae includes about 32 genera and 2500 species that are distributed in tropical and subtropical regions, especially in Southeast Asia [1]. In Korea, members of the Lauraceae family are found in the southern regions and in Jeju Island. In this study, we describe the chemical constituents and activity of *Machilus japonica* extracts. *M. japonica* is an evergreen tree with yellow-green flowers and oval leaves. A few studies on the chemical composition of this plant have resulted in the identification of lignans [2] and terpene compounds [3]. In addition, the biological activities of *M. japonica* extracts such as insecticidal activity [4], elastase inhibition activity [5], and inhibition of matrix metalloproteinase-9 activity [6] have been investigated; however, all of these activities have been identified from extracts of *M. japonica* leaves. Taking a different approach, the aim of this study was to explore the active components of the stem of *M. japonica* and to screen their biological activities.

A total of six flavonoids were isolated and identified from the stem of *M. japonica*. Because many flavonoids have been reported to exhibit antioxidant activities [7], the isolated flavonoids were also evaluated for DPPH radical scavenging activity. low-density lipoprotein (LDL) is susceptible to oxidative damage, and oxidized LDL (oxLDL) plays a key role in the development of atherosclerotic lesions [8]. oxLDL in vessel walls is subjected to rapid uptake by scavenger receptors on monocyte derived macrophages, leading to the formation of foam cells that accumulate cholesterol [9]. Thus, we also evaluated the isolated compounds for their ability to inhibit LDL oxidation. Our results suggest that extracts of *M. japonica* stems and the specific flavonoids found in these extracts may prove useful for preventing or treating hypercholesterolemia and atherosclerosis.

## 2. Results and Discussion

### 2.1. Isolation and Structure Elucidation

**Figure 1:**
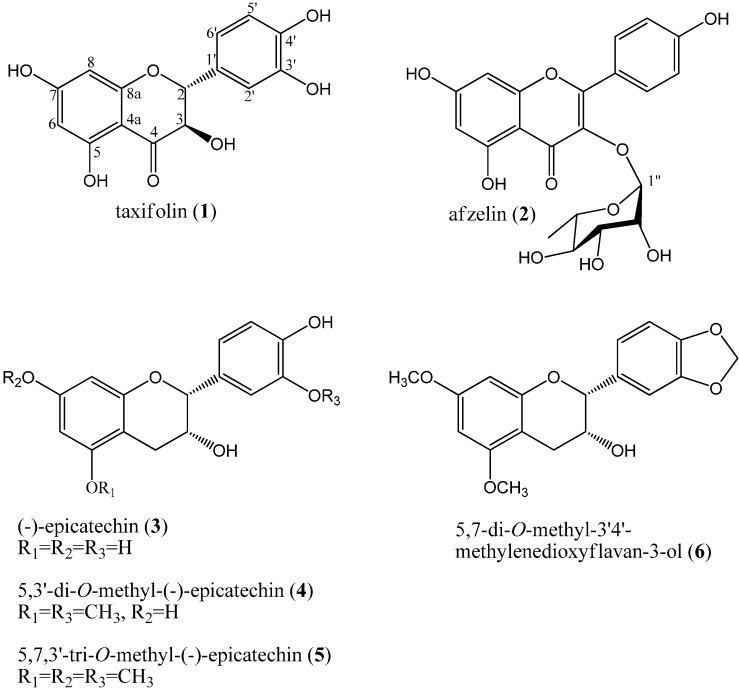
Chemical structures of the six flavonoids isolated from the stem of *M. japonica*.

When the methanol extracts of *M. japonica* stem were resolved on silica gel thin layer chromatograms (TLC), some spots showed UV absorption and yellow colorization when sprayed with 10% H_2_SO_4_ solution and heating, indicating the presence of flavonoids in the extracts. The methanol extract was fractionated into EtOAc, *n*-BuOH, and H_2_O layers through solvent fractionation. Repeated silica gel and ODS c.c. of the EtOAc fraction yielded six purified compounds. The chemical structures of the compounds were determined by ^1^H-NMR, ^13^C-NMR, distortionless enhancement by polarization transfer (DEPT), gradient correlation spectroscopy (gCOSY), gradient heteronuclear single-quantum coherence (gHSQC), and gradient heteronuclear multiple-bond connectivity (gHMBC) analysis as well as IR and MS spectroscopic data. Compounds **1**–**6** were identified as (+)-taxifolin (**1**), afzelin (**2**), (−)-epicatechin (**3**), 5,3'-di-*O*-methyl-(−)-epicatechin (**4**), 5,7,3'-tri-*O*-methyl-(−)-epicatechin (**5**), and 5,7-di-*O*-methyl-3',4'-methylenedioxyflavan-3-ol (**6**) based on physical and spectroscopic evidence and confirmed through comparison with published data (Figure 1).

Compound **1** was isolated as a yellow amorphous powder and showed IR absorbance bands for hydroxyl (3420 cm^−1^), conjugated ketone (1620 cm^−1^), and aromatic (1520 cm^−1^) groups. The molecular weight was determined to be 304 g/mol based on the pseudomolecular ion peak *m*/*z* 305 [M + H]^+^ in the positive FAB/MS spectrum. The ^1^H-NMR spectrum exhibited three olefin proton signals of a 1,2,4-trisubstituted benzene ring at δ_H_ 6.95 (1H, d, *J* = 2.0 Hz), δ_H_ 6.83 (1H, dd, *J* = 8.0, 2.0 Hz), and δ_H_ 6.79 (1H, d, *J* = 8.0 Hz) and two olefin methine proton signals of a 1,2,3,5-tetrasubstituted benzene ring at δ_H_ 5.90 (1H, d, *J* = 2.0 Hz) and δ_H_ 5.86 (1H, d, *J* = 2.0 Hz). Two oxygenated methine proton signals at δ_H_ 4.89 (1H, d, *J* = 11.6 Hz) and δ_H_ 4.49 (1H, d, *J* = 11.6 Hz), which were coupled with each other, were observed. From the *J* value (11.6 Hz) between the two oxygenated methine proton signals, compound **1** was deduced to be a 2,3-*trans* configured flavanol. In the ^13^C-NMR spectrum, there were 15 carbon signals including one ketone signal at δ_C_ 198.1 (C-4). The twelve carbon signals were due to two benzene rings composed of five oxygenated olefin quaternary carbon signals at δ_C_ 168.5 (C-7), δ_C_ 165.1 (C-5), δ_C_ 164.2 (C-8a), δ_C_ 146.9 (C-4'), and δ_C_ 146.1 (C-3'), two olefin quaternary carbon signals at δ_C_ 129.6 (C-1') and δ_C_ 100.4 (C-4a), and five olefin methine carbon signals at δ_C_ 120.7 (C-6'), δ_C_ 115.9 (C-2'), δ_C_ 115.7 (C-5'), δ_C_ 97.2 (C-6), and δ_C_ 96.1 (C-8). The multiplicity of each carbon was determined by DEPT. Two oxygenated methine carbon signals at δ_C_ 85.0 (C-2) and δ_C_ 73.5 (C-3) were also observed. The combination of ^1^H, ^13^C-NMR, and specific rotation (+41.0°) data led to the conclusion that compound **1** was (2*R*,3*R*)-5,7,3',4'-tetrahydroxyflavanonol, (+)-taxifolin [10,11].

Compound **2** was isolated as a yellow amorphous powder and showed IR absorbance bands for hydroxyl (3280 cm^−1^), conjugated ketone (1655 cm^−1^), and aromatic (1615 cm^−1^) groups. The molecular weight was determined to be 432 g/mol based on the pseudomolecular ion peak *m*/*z* 431 [M−H]^−^ in the negative ESI/MS spectrum. In the ^1^H-NMR spectrum, compound **2** showed proton signals of a para-disubstituted benzene ring at δ_H_ 7.75 (2H, br. d, *J* = 8.8 Hz) assigned for H-2', 6' and δ_H_ 6.92 (2H, br. d, *J* = 8.8 Hz) assigned for H-3', 5', and one 1,2,3,5-tetrasubstituted benzene ring at δ_H_ 6.37 (1H, d, *J* = 2.0 Hz) and δ_H_ 6.19 (1H, d, *J* = 2.0 Hz). In addition, there was one doublet hemiacetal proton signal at δ_H_ 5.36 with a coupling constant of 1.6 Hz, one oxygenated proton signal at δ_H_ 4.21 (1H, dd, *J* = 2.8, 1.6 Hz) along with additional oxygenated methine proton signals in the region from δ_H_ 3.93 to δ 3.68, and one methyl proton signal δ_H_ 0.91 (3H, d, *J* = 5.6 Hz) in the upfield shift, indicating the presence of a 6-deoxysugar. The ^13^C-NMR spectrum showed 21 carbon signals indicating that compound **2** was a flavonoid monoglycoside. The carbon signals observed included one ketone signal at δ_C_ 178.0 (C-4), six oxygenated olefin quaternary signals at δ_C_ 164.3 (C-5), δ_C_ 161.1 (C-4a), δ_C_ 160.0 (C-4'), δ_C_ 157.2 (C-2), δ_C_ 157.0 (C-7), and δ_C_ 134.6 (C-3), two olefin quaternary signals at δ_C_ 121.1 (C-1') and δ_C_ 108.4 (C-8a), and six olefin methine signals at δ_C_ 130.4 (C-2', 6'), δ_C_ 115.0 (C-3', 5'), δ_C_ 98.4 (C-6), and δ_C_ 93.3 (C-8). The chemical shift and multiplicity data led us to determine that compounds **2** was a trihydroxyflavonol glycoside with a deoxyhexose moiety. The carbon resonance due to the 6-deoxysugar moiety including an anomer carbon signal at δ_C_ 102.0 (C-1"), four oxygenated methine carbon signals at δ_C_ 71.7 (C-4"), δ_C_ 70.7 (C-3"), δ_C_ 70.6 (C-5"), and δ_C_ 70.5 (C-2"), and one methyl carbon signal at δ_C_ 16.3 (C-6") indicated that the sugar was l-rhamnopyranose. The configuration of the anomer carbon was determined to be α based on the coupling constant of the anomer proton signal (*J* = 1.6 Hz) in the ^1^H-NMR spectrum and the chemical shift of the anomer carbon signal (δ_C_ 102.0) in the ^13^C-NMR spectrum. To determine the position of the α-l-rhamnopyranosyl moiety, a gHMBC experiment was conducted. The gHMBC spectrum showed that the anomer proton signal (δ_H_ 5.36, H-1") was correlated with the oxygenated olefin quaternary carbon signal (δ_C_ 134.6, C-3), suggesting the presence of a glycosidic bond at C-3. Based on comparison of these data with those in the reported literature [12,13], compound **2** was identified as kaempferol-3-*O*-α-l-rhamnopyranoside, also known as afzelin.

Compound **3** was isolated as a yellow amorphous powder and showed IR absorbance bands for hydroxyl (3450 cm^−1^) and aromatic (1624 cm^−1^) groups. The molecular weight was determined to be 290 g/mol based on the molecular ion peak *m*/*z* 290 [M]^+^ in the EI/MS spectrum. The ^1^H-NMR spectrum showed three olefin methine proton signals at δ_H_ 6.96 (1H, d, *J* = 1.6 Hz), δ_H_ 6.78 (1H, dd, *J* = 8.0, 1.6 Hz), and δ_H_ 6.75 (1H, d, *J* = 8.0 Hz) due to a 1,2,4-trisubstituted benzene ring, and two olefin methine proton signals at δ_H_ 5.93 (1H, d, *J* = 1.6 Hz) and δ_H_ 5.91 (1H, d, *J* = 1.6 Hz) due to a 1,2,3,5-tetrasubstituted benzene ring. Two oxygenated methine proton signals at δ_H_ 4.80 (1H, br. s) and δ_H_ 4.16 (1H, m) and one methylene proton signal at δ_H_ 2.85 (1H, dd, *J* = 16.8, 4.8 Hz) and δ_H_ 2.73 (1H, dd, *J* = 16.8, 2.8 Hz) were detected in the upfield shift. Coupling patterns of the oxygenated methine protons of the broad singlet and multiplet led us to assume that compound **3** was a 2,3-*cis* configured flavan-3-ol. A total of 15 carbon signals were observed in the ^13^C-NMR spectrum. From the downfield shift, 12 carbon signals of two benzene rings were observed; five oxygenated olefin quaternary signals at δ_C_ 157.9 (C-5), δ_C_ 157.5 (C-7), δ_C_ 157.3 (C-8a), δ_C_ 145.8 (C-4'), and δ_C_ 145.7 (C-3'), two olefin quaternary signals at δ_C_ 132.2 (C-1') and δ_C_ 100.0 (C-4a), and five olefin methine signals at δ_C_ 119.3 (C-6'), δ_C_ 115.8 (C-5'), δ_C_ 115.2 (C-2'), δ_C_ 96.3 (C-6), and δ_C_ 95.8 (C-8). In the upfield shift, two oxygenated methine carbon signals at δ_C_ 79.8 (C-2) and δ_C_ 67.4 (C-3), and one methylene carbon signal at δ_C_ 29.2 (C-4) were observed. Consequently, this compound was identified as (2*R*,3*R*)-5,7,3',4'-tetrahydroxyflavan-3-ol, (−)-epicatechin, which was further confirmed through a comparison of spectroscopic data, including the specific rotation value ([α]_D_ −57.0°), with data reported in the literature [14].

Compound **4** was obtained as a yellow powder and a molecular ion peak [M]^+^ was observed at *m*/*z* 318 in the EI/MS spectrum. The IR spectrum showed absorbance bands for hydroxyl (3366 cm^−1^) and aromatic (1622 cm^−1^) groups. The ^1^H-NMR spectrum showed three olefin methine proton signals at δ_H_ 7.11 (1H, d, *J* = 2.0 Hz), 6.88 (1H, dd, *J* = 8.4, 2.0 Hz) and 6.77 (H, d, *J* = 8.4 Hz) of a 1,2,4-trisubstituted benzene ring and two olefin methine proton signals at δ_H_ 6.03 (1H, d, *J* = 2.4 Hz) and 6.00 (1H, d, *J* = 2.4 Hz) with evidence of long range coupling (^4^*J*) consistent with a 1,2,3,5-tetrasubstituted benzene ring. Two oxygenated methine proton signals at δ_H_ 4.88 (1H, br. s) and 4.17 (1H, m), two methoxy proton signals at δ_H_ 3.85 (3H, s) and 3.75 (3H, s), and methylene proton signals at δ_H_ 2.85 (1H, dd, *J* = 17.2, 4.8 Hz) and 2.73 (1H, dd, *J* = 17.2, 2.8 Hz) were also observed. Thus, compound **4** appeared to be very similar to compound **3** with the exception of two methoxy groups. In the ^13^C-NMR spectrum, 17 carbon signals were detected including two methoxy groups. In the downfield shift, there were five oxygenated olefin quaternary carbon signals at δ_C_ 160.5 (C-7), δ_C_ 158.0 (C-5), δ_C_ 157.0 (C-8a), δ_C_ 148.6 (C-3'), and δ_C_ 147.0 (C-4'), two olefin quaternary carbon signals at δ_C_ 132.2 (C-1') and δ_C_ 100.9 (C-4a), and five olefin methine carbon signals at δ_C_ 120.5 (C-6'), δ_C_ 115.6 (C-2'), δ_C_ 111.8 (C-5'), δ_C_ 96.7 (C-6), and δ_C_ 92.8 (C-8) due to two benzene rings. In addition, two oxygenated methine carbon signals at δ_C_ 80.0 (C-2) and δ_C_ 67.4 (C-3) and one methylene carbon signal at δ_C_ 29.3 (C-4) were observed. Therefore, compound **4** was determined to be a (−)-epicatechin with two methoxy groups. Based on the correlation of the two methoxy signals at δ_H_ 3.85 (3H, s) and 3.75 (3H, s) with the two oxygenated olefin quaternary carbon signals at δ_C_ 158.0 (C-5) and δ_C_ 148.6 (C-3') in the gHMBC spectrum, compound **4** was identified as 5,3'-di-*O*-methyl-(−)-epicatechin, which was confirmed by comparison of the spectroscopic data with values reported in the literature [15].

Compound **5** was obtained as colorless needles and a molecular ion peak [M]^+^ was observed at *m*/*z* 332 in the EI/MS spectrum. The IR spectrum showed absorbance bands for hydroxyl (3412 cm^−1^) and aromatic (1616 cm^−1^) groups. The ^1^H-NMR and ^13^C-NMR spectra of compounds **4** and **5** were analogous, showing (−)-epicatechin skeleton signals. Due to the *m*/*z* of 332 and observation of three methoxy groups, compound **5** was presumed to be (−)-epicatechin-tri-*O*-methylate. To confirm the position of the methoxy groups, a gHMBC experiment was conducted. Judging by the correlation between the three methoxy proton signals at δ_H_ 3.69 (6H, s) and 3.66 (3H, s) with three oxygenated olefin quaternary carbon signals at δ_C_ 160.0 (C-7), δ_C_ 159.6 (C-5), and δ_C_ 148.3 (C-3') in the gHMBC spectrum, compound **5** was identified as 5,7,3'-tri-*O*-methyl-(−)-epicatechin, which was confirmed by comparison with spectroscopic data in the literature [15].

Compound **6** was obtained as a white amorphous powder and a molecular ion peak [M]^+^ was observed at *m*/*z* 330 in the EI/MS spectrum. The IR spectrum showed absorbance bands of hydroxyl (3408 cm^−1^) and aromatic (1591 cm^−1^) groups. The ^1^H-NMR spectrum showed that compound **6** had a similar pattern to compound **4** apart from one dioxymethylene signal at δ_H_ 5.94 (2H, s). In the ^13^C-NMR spectrum, 18 carbon signals were detected including two methoxy groups (δ_C_ 55.4, 55.3) and one dioxymethylene (δ_C_ 101.0) signal. Based on the correlation between the two oxygenated olefin quaternary carbon signals at δ_C_ 147.8 (C-3') and δ_C_ 147.3 (C-4') and the dioxymethlylene proton signal at δ_H_ 5.94 (2H, s), the two oxygenated olefin quaternary carbon signals at δ_C_ 159.6 (C-5) and δ_C_ 159.2 (C-7), and two methoxy proton signals at δ_H_ 3.76 (3H, s) and δ_H_ 3.74 (3H, s), compound **6** was determined as 5,7-di-*O*-methyl-3',4'-methylenedioxyflavan-3-ol, which was confirmed by comparison with spectroscopic data in the literature [16].

### 2.2. Evaluation of Radical Scavenging Activity and Inhibitory Effect on LDL Oxidation

Compounds **1** and **3**–**6** exhibited DPPH radical scavenging with IC_50_ values of 0.16, 0.21, 0.17, 0.15, and 0.07 mM, respectively, which were equivalent with that of ascorbic acid (0.18 mM) (Table 1). The lack of scavenging activity by compound **2** was speculated to be due to the glucosyl moiety on the C ring [17]. Compound **6** exhibited the highest antioxidant activity, which we attributed to the dioxymethylene moiety on the B ring.

**Table 1:** 1,1-diphenyl-2-picrylhydrazyl (DPPH) radical scavenging activity of flavonoids isolated from the stems of *Machilus japonica*. *^a^* The IC_50_ value of each compound was defined as the concentration (mM) that resulted in 50% DPPH scavenging activity. The results are averages of three independent experiments.

Compound	1	2	3	4	5	6	Ascorbic Acid
IC_50_ (mM) *^a^*	0.16	–	0.21	0.17	0.15	0.07	0.18

Highly reactive molecules called free radicals can cause tissue damage by reacting with polyunsaturated fatty acids in cellular membranes, nucleotides in DNA, and critical sulfhydryl bonds in proteins. In addition to cellular damage, cataract formation, photodermatoses, aging, and inflammatory diseases such as arthritis are associated with free radicals [18]. Therefore, the search for compounds from natural sources that can protect against free radicals by endogenous and exogenous antioxidants is of special significance for human health.

Antioxidants act by donating hydrogen atoms to lipid radicals. Radicals obtained from antioxidants with molecular structures such as phenols are stable species that can halt the oxidation chain reaction [19]. To determine whether these compounds might be effective in the development of hypercholesterolemic or antiatherogenic agents, their potential for inhibiting LDL oxidation was evaluated.

Compounds **1**, **3**, and **5** demonstrated LDL antioxidant activity with IC_50_ values of 2.8, 7.1, and 4.6 µM, respectively (Table 2). Compound **1** was similar to the positive control, BHT, which had an IC_50_ value of 1.9 µM, while compounds **3** and **5** also showed significant activity. Compound **4** exhibited a low level of LDL antioxidant activity with an IC_50_ value of 79.1 µM. Compounds **2** and **6** were not effective as LDL antioxidants, which was attributed to the glucosyl moiety at C-3 for compound **2** and steric hindrance of the hydroxyl groups at C-3' and C-4' by dioxymethylene moieties in compound **6**.

**Table 2:** Inhibitory effects of flavonoids isolated from the stems of *Machilus japonica* on low-density lipoprotein (LDL) oxidation. *^a^* The IC_50_ value of each compound was defined as the concentration (μM) that resulted in 50% inhibition of LDL oxidation. The results are averages of three independent experiments, and the data are expressed as the mean ± SD.

Compound	Inhibition Effect (%)				IC_50_ (µM) *^a^*
	5 µM	10 µM	40 µM	80 µM	
**1**	90.1 ± 0.0	95.8 ± 0.6	100.4 ± 0.0	104.2 ±1.5	2.8 ± 0.5
**2**	–	–	–	3.0 ± 1.8	>50
**3**	23.1 ± 2.6	93.6 ± 1.6	99.0 ± 0.2	104.0 ± 0.0	7.1 ± 1.1
**4**	–	–	–	51.8 ± 0.2	>50
**5**	58.9 ± 0.7	65.3 ± 1.4	72.9 ± 0.5	84.2 ± 0.7	4.6 ± 0.8
**6**	–	–	–	13.7 ± 0.2	>50
**BHT**	–	–	–	–	1.9 ± 0.4

LDL oxidation is regarded as a key step in the formation of atherosclerotic lesions [20,21]. Experimental evidence demonstrating an association between oxLDL cholesterol and both the presence of atherosclerotic lesions and progression of carotid artery atherosclerosis support this hypothesis [22,23]. Vitamin E, one of the most popular natural antioxidants, inhibits atherogenesis by inhibiting LDL oxidation (IC_50_: 2.4 μM) [24]. Several other effective natural dietary antioxidants comprising phenolic compounds and carotenoids have been identified as well, in addition to vitamins and enzymes [25]. The inhibitory activity of compounds **1**, **3**, and **5** was very similar to that of the well-known antioxidant BHT (IC_50_: 1.9 μM), and showed significant antioxidant capacity relative to several other naturally-occurring antioxidants such as nectandrin B (IC_50_: 4.1 μM) from tabu (*Machilus thunbergii*) [26] and (+)-lariciresinol (IC_50_: 11.9 μM) from Rousa dogwood (*Cornus kousa*) [27]. Therefore, the flavonoids isolated from *Machilus japonica* may be a good natural source of antiatherogenic agents.

## 3. Experimental Section

### 3.1. Plant Materials

Dried stems of *Machilus japonica* were supplied by GFC Co., Ltd., Suwon, Korea in January 2010, and were identified by Dae-Keun Kim, College of Pharmacy, Woosuk University, Jeonju, Korea. A voucher specimen (KHU2010-0103) has been reserved at the Laboratory of Natural Product Chemistry, Kyung Hee University, Yongin, Korea.

### 3.2. General Experimental Procedures

Melting points were determined using a Fisher-John’s Melting Point apparatus (Fisher Scientific, Miami, FL, USA) with a microscope and the values obtained were uncorrected. Optical rotations were measured using a JASCO P-1010 digital polarimeter (Tokyo, Japan). NMR spectra were recorded on a 400 MHz FT-NMR spectrometer (Varian Inova AS 400, Palo Alto, CA, USA). IR spectra were obtained from a Perkin Elmer Spectrum One FT-IR spectrometer (Buckinghamshire, England). FAB-MS data were recorded on a JEOL JMS-700 (Tokyo, Japan), EI-MS on a JEOL JMSAX 505-WA (Tokyo, Japan), and ESI-MS on a Finnigan LCQ Advantage spectrometer (Thermo Scientific, Waltham, MD, USA). The UV lamp used was a Spectroline Model ENF-240 C/F (Spectronics Corporation, Westbury, NY, USA). Kiesel gel 60 silica gel resin was used for column chromatography (c.c.) (Merck, Darmstadt, Germany) and the ODS was a LiChroprep RP-18 (Merck). TLC analysis was carried out using Kiesel gel 60 F_254_ and RP-18 F_254S_ (Merck). Deuterated solvents were purchased from Merck Co. Ltd. and Sigma Aldrich Co. Ltd. (St. Louis, MO, USA).

### 3.3. Isolation of Flavonoids from the EtOAc Fraction Obtained from M. japonica Stems

Dried and pulverized stems (9 kg) of *M. japonica* were extracted with 80% aqueous MeOH (25 L × 3) at room temperature for 24 h. Concentrated MeOH extracts were suspended in H_2_O (3 L) and then successively extracted with ethyl acetate (EtOAc, 3 L × 4) and *n*-butanol (*n*-BuOH, 2.4 L × 3). The solutions were again concentrated to produce an EtOAc fraction (MJE, 56 g), *n*-BuOH fraction (MJB, 75 g), and H_2_O fraction (MJW, 229 g). The EtOAc fraction extract was applied to silica gel (SiO_2_) c.c. (φ 10 cm × 16 cm), eluted with chloroform (CHCl_3_)-MeOH (15:1 → 12:1 → 10:1 →8:1 → 5:1 → 1:1, 3.2 L each), and monitored by TLC to produce 18 fractions (MJE-1 to MJE-18). Fraction MJE-2 [7.4 g, elution volume/total volume (Ve/Vt) 0.03–0.08] was applied to SiO_2_ c.c. (φ 7 cm × 12 cm) and eluted with *n*-hexane-EtOAc (8:1 → 4:1 → 1:1, 4 L each) to produce 16 fractions (MJE-2-1 to MJE-2-16). Among these, fraction MJE-2-10 (490 mg, Ve/Vt 0.42–0.51) was separated by ODS c.c. (φ 3.5 cm × 4.5 cm) using acetone-H_2_O (1:3, 3L → 1:1, 4L) as the elution solvent to obtain compound **6** [MJE-2-10-5, 17 mg, Ve/Vt 0.45–0.52, TLC (RP-18 F_254S_) R_f_ 0.49, acetone-H_2_O = 3:1]. Fraction MJE-2-13 (512 mg, Ve/Vt 0.75–0.81) was subjected to SiO_2_ c.c. (φ 3.5 cm × 12 cm) with *n*-hexane-CHCl_3_-MeOH (20:10:1, 1 L) as the elution solvent to produce seven fractions (MJE-2-13-1 to MJE-2-13-7). Fraction MJE-2-13-3 (143 mg, Ve/Vt 0.38–0.44) was applied to ODS c.c. (φ 3 cm × 5.5 cm) using MeOH-H_2_O (1:4, 2.8 L) as the elution solvent to yield compound **5** [MJE-2-13-3-10, 28 mg, Ve/Vt 0.75–0.77, TLC (RP-18 F_254S_) R_f_ 0.75, acetone-H_2_O = 2:1]. Fraction MJE-3 (3.8 g, Ve/Vt 0.08–0.13) was applied to SiO_2_ c.c. (φ 7 cm × 12 cm) and eluted with *n*-hexane-EtOAc (10:1, 20 L) to produce 20 fractions (MJE-3-1 to MJE-3-20). Fraction MJE-3-17 (120 mg, Ve/Vt 0.66–0.68) was separated by ODS c.c. (φ 3.0 cm × 5.0 cm) using acetone-H_2_O (1:5, 2.2 L) as the elution solvent to obtain compound **4**　[MJE-3-17-4, 17 mg, Ve/Vt 0.45–0.52, TLC (RP-18 F_254S_) R_f_ 0.46, acetone-H_2_O = 1:1]. Fraction MJE-11 (2.4 g, Ve/Vt 0.50–0.55) was applied to ODS c.c. (φ 3.5 cm × 7 cm) and eluted with MeOH-H_2_O (1:5 → 1:3 → 1:1 → 3:1 → 10:1, 1.6 L each) to produce 18 fractions (MJE-11-1 to MJE-11-18). Fraction MJE-11-6 (123 mg, Ve/Vt 0.25–0.37) was subjected to ODS c.c. (φ 3 cm × 5 cm) and eluted with MeOH-H_2_O (1:2, 1.6 L) to produce eight fractions (MJE-11-6-1 to MJE-11-6-8). Fraction MJE-11-6-1 (49 mg, Ve/Vt 0.00–0.07) was subjected to SiO_2_ c.c. (φ 2 cm × 11 cm) and eluted with CHCl_3_-MeOH-H_2_O (10:3:1, 1.1 L) to yield compound **3** [MJE-11-6-1-4, 9.5 mg, Ve/Vt 0.31–0.76, TLC (RP-18 F_254S_) R_f_ 0.69, acetone-H_2_O = 1:1]. Fraction MJE-11-6-3 (33 mg, Ve/Vt 0.18–0.24) was subjected to ODS c.c. (φ 2 cm × 7 cm) and eluted with acetone-H_2_O (1:3, 350 mL) to yield compound **1** [MJE-11-6-3-3, 18 mg, Ve/Vt 0.46–0.99, TLC (RP-18 F_254S_) R_f_ 0.50, acetone-H_2_O = 1:1]. Finally, fraction MJE-11-11 (180 mg, Ve/Vt 0.50–0.53) was subjected to SiO_2_ c.c. (φ 3 cm × 13 cm) and eluted with CHCl_3_-MeOH-H_2_O (13:3:1, 1.1 L) to yield compound **2** [MJE-11-11-6, 9.6 mg, Ve/Vt 0.31–0.76, TLC (F_254S_) R_f_ 0.51, CHCl_3_-MeOH-H_2_O = 7:3:1].

*Compound **1** ((+)-taxifolin).* Yellow amorphous powder (MeOH); m.p. 231 °C; [α]_D_ +42.0° (*c* = 0.50, MeOH); IR (CaF_2_ plate, ν) 3420, 2931, 2885, 1620, 1520, 1470, 1360, 1265, 1165 cm^−1^; positive FAB-MS *m*/*z* 305 [M + H]^+^; ^1^H-NMR (400 MHz, CD_3_OD, δ) 6.95 (1H, d, *J* = 2.0 Hz, H-2'), 6.83 (1H, dd, *J* = 8.0, 2.0 Hz, H-6'), 6.79 (1H, d, *J* = 8.0 Hz, H-5'), 5.90 (1H, d, *J* = 2.0 Hz, H-8), 5.86 (1H, d, *J* = 2.0 Hz, H-6), 4.89 (1H, d, *J* = 11.6 Hz, H-2), 4.49 (1H, d, *J* = 11.6 Hz, H-3); ^13^C-NMR (100 MHz, CD_3_OD, δ) 198.1 (C-4), 168.5 (C-7), 165.1 (C-5), 164.2 (C-8a), 146.9 (C-4'), 146.1 (C-3'), 129.6 (C-1'), 120.7 (C-6'), 115.9 (C-2'), 115.7 (C-5'), 100.4 (C-4a), 97.2 (C-6), 96.1 (C-8), 85.0 (C-2), 73.5 (C-3).

*Compound **2** (afzelin).* Yellow amorphous powder (MeOH); m.p. 173 °C; [α]_D_−184.0° (*c* = 0.10, MeOH); IR (CaF_2_ plate, ν) 3280, 2977, 2937, 1655, 1615, 1500, 1450, 1365 cm^−1^; ESI-MS *m*/*z* 431 [M − H]^−^; ^1^H-NMR (400 MHz, CD_3_OD, δ) 7.75 (2H, br. d, *J* = 8.8 Hz, H-2', 6'), 6.92 (2H, br. d, *J* = 8.8 Hz, H-3', 5'), 6.37 (1H, d, *J* = 2.0 Hz, H-8), 6.19 (1H, d, *J* = 2.0 Hz, H-6), 5.36 (1H, d, *J* = 1.6 Hz, H-1"), 4.21 (1H, dd, *J* = 2.8, 1.6 Hz, H-2"), 3.93–3.68 (3H, m, H-3", 4", 5"), 0.91 (3H, d, *J* = 5.6 Hz, H-6"); ^13^C-NMR (100 MHz, CD_3_OD, δ) 178.0 (C-4), 164.3 (C-5), 161.1 (C-4a), 160.0 (C-4'), 157.2 (C-2), 157.0 (C-7), 134.6 (C-3), 130.4 (C-2', 6'), 121.1 (C-1'), 115.0 (C-3', 5'), 108.4 (C-8a), 102.0 (C-1"), 98.4 (C-6), 93.3 (C-8), 71.7 (C-4"), 70.7 (C-3"), 70.6 (C-5"), 70.5 (C-2"), 16.3 (C-6").

*Compound **3** ((−)-epicatechin).* Yellow amorphous powder (MeOH); m.p. 243 °C; [α]_D_ −57.0° (*c* = 0.50, MeOH); IR (KBr, ν) 3450, 2935, 1525, 1469, 1438, 1263, 1184 cm^−1^; EI-MS *m*/*z* 290 [M]^+^; ^1^H-NMR (400 MHz, CD_3_OD, δ) 6.96 (1H, d, *J* = 1.6 Hz, H-2'), 6.78 (1H, dd, *J* = 8.0, 1.6 Hz, H-6'), 6.75 (1H, d, *J* = 8.0 Hz, H-5'), 5.93 (1H, d, *J* = 1.6 Hz, H-8), 5.91 (1H, d, *J* = 1.6 Hz, H-6), 4.80 (1H, br. s, H-2), 4.16 (1H, m, H-3), 2.85 (1H, dd, *J* = 16.8, 4.8 Hz, H-4β), 2.73 (1H, dd, *J* = 16.8, 2.8 Hz, H-4α); ^13^C-NMR (100 MHz, CD_3_OD, δ) 157.9 (C-5), 157.5 (C-7), 157.3 (C-8a), 145.8 (C-4'), 145.7(C-3'), 132.2 (C-1'), 119.3 (C-6'), 115.8 (C-5'), 115.2 (C-2'), 100.0 (C-4a), 96.3 (C-6), 95.8 (C-8), 79.8 (C-2), 67.4 (C-3), 29.2 (C-4).

*Compound **4** (5,3*'*-di-O-methyl-(−)-epicatechin).* Yellow powder (CHCl_3_); m.p. 267 °C; [α]_D_ −75.0° (*c* = 0.50, MeOH); IR (CaF_2_ plate, ν) 3366, 2956, 2862, 2340, 1622, 1503, 1373, 1270, 1145 cm^−1^; EI-MS *m*/*z* 318 [M]^+^; ^1^H-NMR (400 MHz, CDCl_3_, δ) 7.11 (1H, d, *J* = 2.0 Hz, H-2'), 6.88 (1H, dd, *J* = 8.4, 2.0 Hz, H-6'), 6.77 (1H, d, *J* = 8.4 Hz, H-5'), 6.03 (1H, d, *J* = 2.4 Hz, H-8), 6.00 (1H, d, *J* = 2.4 Hz, H-6), 4.88 (1H, br. s, H-2), 4.17 (1H, m, H-3), 3.85 (3H, s, O-Me), 3.75 (3H, s, O-Me), 2.85 (1H, dd, *J* = 17.2, 4.8 Hz, H-4β), 2.73 (1H, dd, *J* = 17.2, 2.8 Hz, H-4α); ^13^C-NMR (100 MHz, CD_3_OD, δ) 160.5 (C-7), 158.0 (C-5), 157.0 (C-8a), 148.6 (C-3'), 147.0 (C-4'), 132.2 (C-1'), 120.5 (C-6'), 115.6 (C-2'), 111.8 (C-5'), 100.9 (C-4a), 96.7 (C-6), 92.8 (C-8), 80.0 (C-2), 67.4 (C-3), 56.3 (O-Me), 55.8 (O-Me), 29.3 (C-4).

*Compound **5** (5,7,3*'*-tri-O-methyl-(−)-epicatechin).* Colorless needles (C_5_H_5_N); m.p. 139 °C; [α]_D_ −62° (*c* = 1.22, acetone); IR (CaF_2_ plate, ν) 3412, 2933, 2838, 1616, 1590, 1519, 1493, 1271, 1145 cm^−1^; EI-MS *m*/*z* 332 [M]^+^, ^1^H-NMR (400 MHz, C_5_D_5_N, δ) 7.37 (1H, dd, *J* = 8.0, 1.6 Hz, H-6′), 7.28 (1H, d, *J* = 8.0 Hz, H-5'), 7.19 (1H, d, *J* = 1.6 Hz, H-2'), 6.52 (1H, br. s, H-6), 6.32 (1H, br. s, H-8), 5.25 (1H, br. s, H-2), 4.62 (1H, m, H-3), 3.69 (6H, s, O-Me), 3.66 (3H, s, O-Me), 3.30 (1H, dd, *J* = 16.8, 4.4 Hz, H-4β), 3.15 (1H, dd, *J* = 16.8, 2.8 Hz, H-4α); ^13^C-NMR (100 MHz, C_5_D_5_N, δ) 160.0 (C-7), 159.6 (C-5), 156.8 (C-8a), 148.3 (C-3'), 147.7 (C-4'), 131.4 (C-1'), 120.6 (C-6'), 116.0 (C-2'), 112.1 (C-5'), 102.0 (C-4a), 94.2 (C-6), 91.9 (C-8), 79.9 (C-2), 66.2 (C-3), 55.7 (O-Me), 55.2 (O-Me × 2), 29.5 (C-4).

*Compound **6** (5,7-di-O-methyl-3*'*,4*'*-methylenedioxyflavan-3-ol).* White amorphous powder (CHCl_3_); m.p. 158 °C; [α]_D_ −34° (*c* = 0.20, CHCl_3_); IR (CaF_2_ plate, ν) 3408, 2920, 2836, 2331, 1591, 1537, 1464, 1252, 1145 cm^−1^; EI-MS *m*/*z* 330 [M]^+^; ^1^H-NMR (400 MHz, CDCl_3_, δ) 7.02 (1H, br. s, H-2'), 6.93 (1H, br. d, *J* = 7.2 Hz, H-6'), 6.82 (1H, d, *J* = 7.2 Hz, H-5'), 6.15 (1H, br. s, H-8), 6.09 (1H, br. s, H-6), 5.94 (2H, s, -O-CH_2_-O-), 4.90 (1H, br. s, H-2), 4.22 (1H, m, H-3), 3.76 (3H, s, O-Me), 3.74 (3H, s, O-Me), 2.83–2.93 (2H, m, H-4α, β); ^13^C-NMR (100 MHz, CD_3_OD, δ) 159.6 (C-5), 159.2 (C-7), 155.1 (C-8a), 147.8 (C-3'), 147.3 (C-4'), 132.1 (C-1'), 119.6 (C-6'), 108.2 (C-5'), 107.1 (C-2'), 101.0 (-O-CH_2_-O-), 93.2 (C-8), 92.1 (C-6), 78.4 (C-2), 66.3 (C-3), 55.4 (O-Me), 55.3 (O-Me), 28.1 (C-4).

### 3.4. DPPH Radical Scavenging Activity

The DPPH radical scavenging activity assay was based on the capacity of a substance to scavenge stable DPPH radicals. Briefly, reaction mixtures containing test samples (100 µL) and an ethanolic DPPH solution (100 µL of 0.06 mM) were placed in 96-well microplates and incubated at 37 °C for 30 min. Absorbance values were measured at 517 nm.

### 3.5. LDL Isolation and Oxidation Assay

Plasma was obtained from fasted healthy normalipidemic volunteers. LDL isolation and TBARS assays were performed as previously described with slight modification [28]. Briefly, an LDL solution (250 μL, 50–100 μg protein) in 10 mM PBS (pH 7.4) was supplemented with 10 μM CuSO_4_. Oxidation was performed in screw-capped 5-mL glass vials at 37 °C in the presence of either compound **1**, **3**, or **5**. After incubation for 4 h the reaction was terminated by the addition of 1 mL 20% TCA. Following precipitation, 1 mL 0.67% TBA in 0.05 N NaOH was added and the mixture was vortexed, after which the final mixture was heated for 5 min at 95 °C, cooled on ice, and centrifuged for 2 min at 1000× *g*. The optical density of MDA generated in the assay was measured at 532 nm. Calibration was performed using an MDA standard prepared from tetramethoxypropane.

## 4. Conclusions

In this study, six flavonoids were isolated from the stem of *M. japonica* for the first time. The isolated compounds were identified as (+)-taxifolin, afzelin, (−)-epicatechin, 5,3'-di-*O*-methyl-(−)-epicatechin, 5,7,3'-tri-*O*-methyl-(−)-epicatechin, and 5,7-di-*O*-methyl-3',4'-methylenedioxyflavan-3-ol based on spectroscopic analyses. The flavonoids showed DPPH radical scavenging activity and inhibitory effects on LDL oxidation. Therefore, extracts of *M. japonica* stems and the flavonoids contained therein may be useful for preventing or treating hypercholesterolemia and atherosclerosis.

## Supplementary Materials

Supplementary Tables can be found at: http://www.mdpi.com/1422-0067/15/9/16418/s1.

## Supplementary Files

Supplementary File 1
