# Supplementary material for: Flavonoids from Machilus japonica Stems and Their Inhibitory Effects on LDL Oxidation
Source: Int J Mol Sci. 2014 Sep 16;15(9):16418–29. doi: 10.3390/ijms150916418 (PMC4200834; doi:10.3390/ijms150916418)

Supplementary Information

Compound **1 (taxifolin)**

^{1}H-NMR spectrum

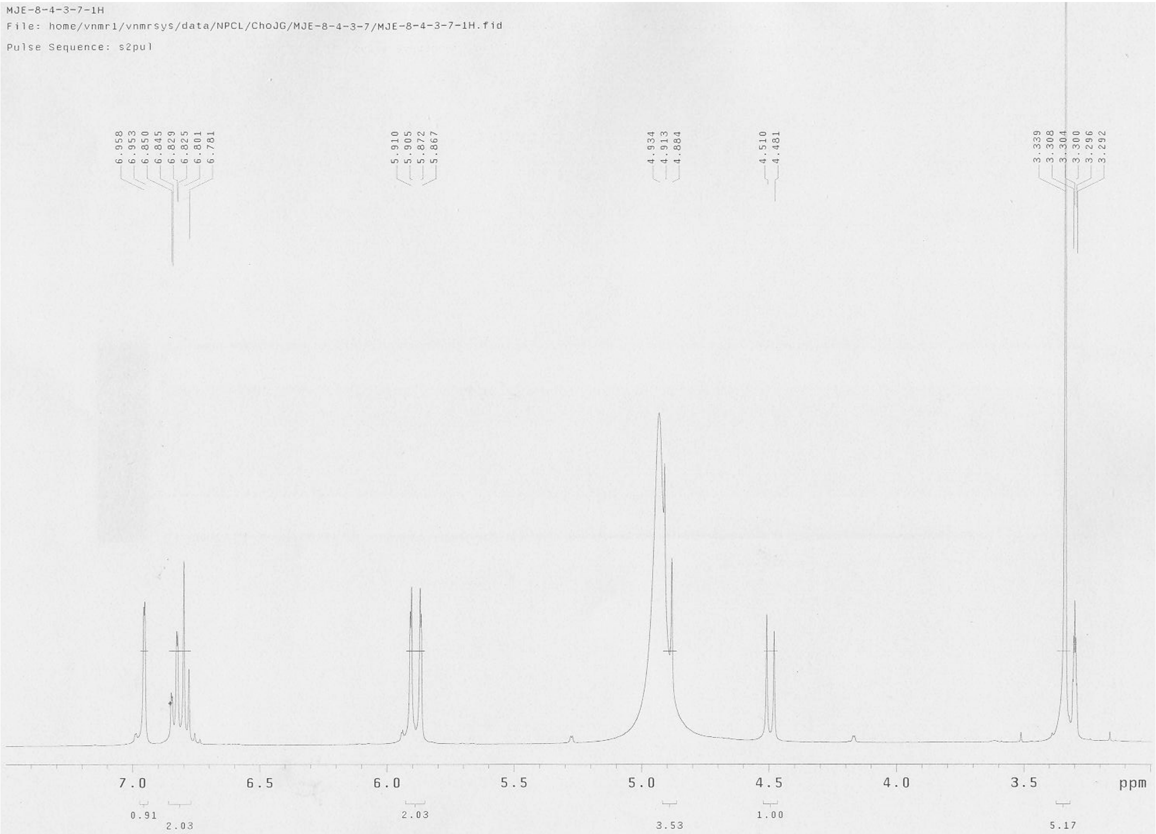

^{13}C-NMR specrrum

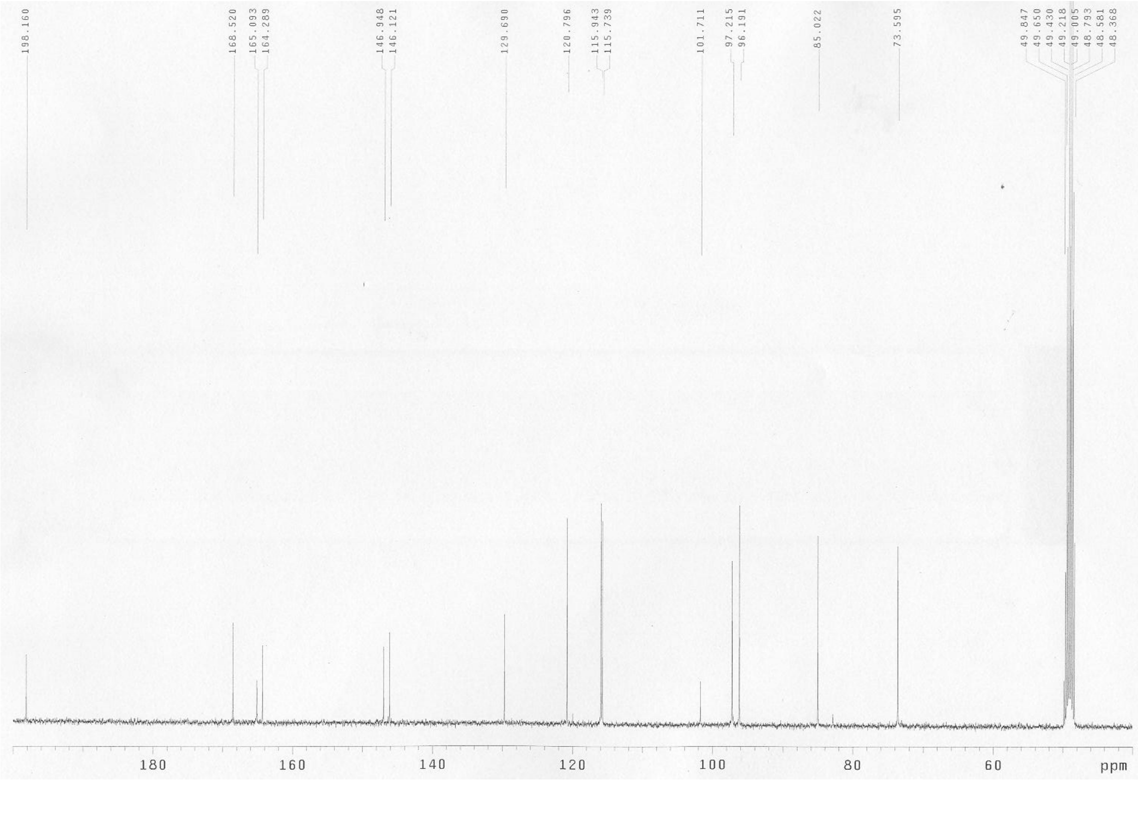

DEPT spectrum

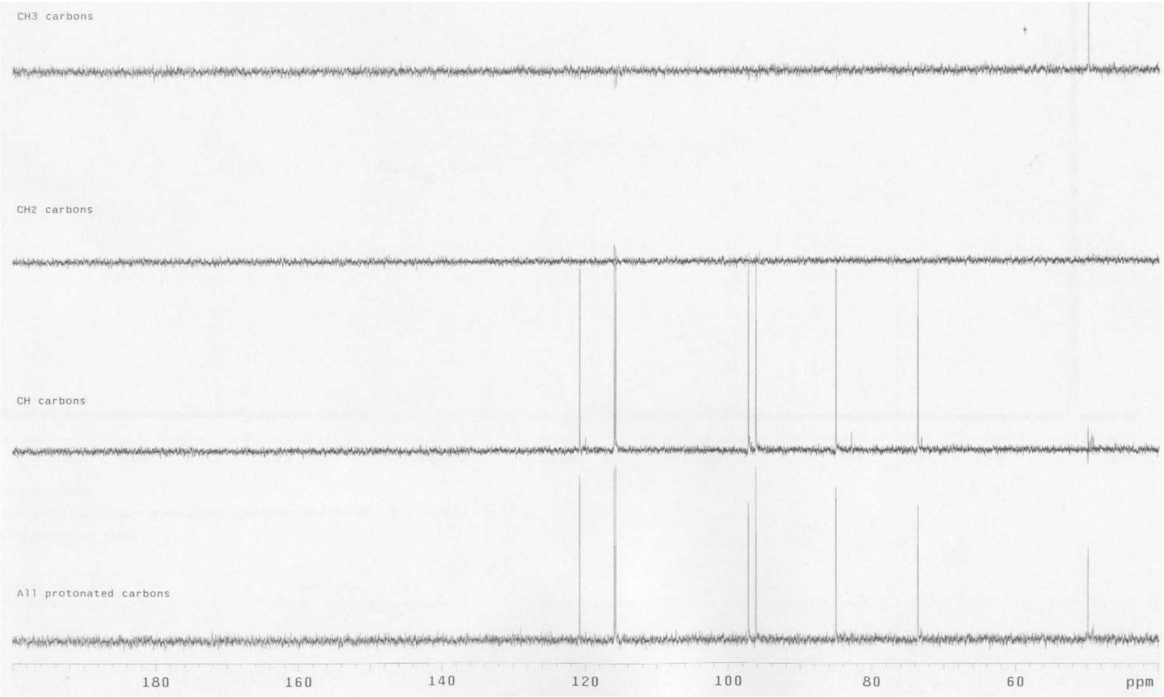

Compound **2 (afzelin)**

^{1}H-NMR spectrum-1

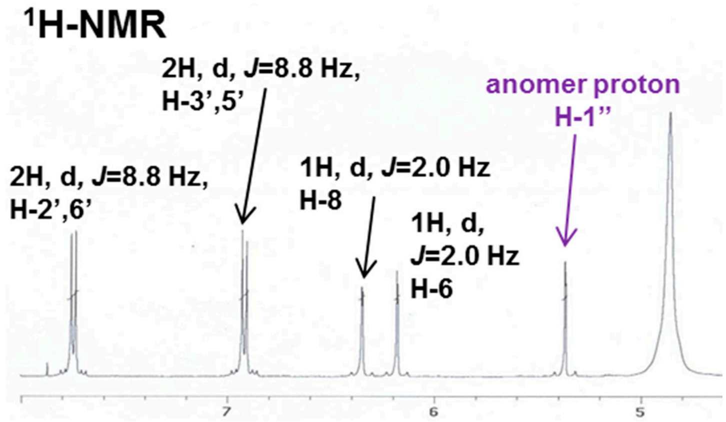

^{1}H-NMR spectrum-2

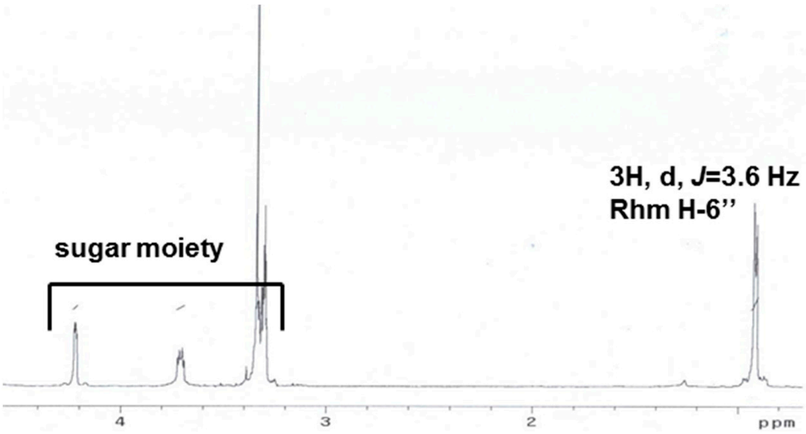

^{13}C-NMR specrrum-1

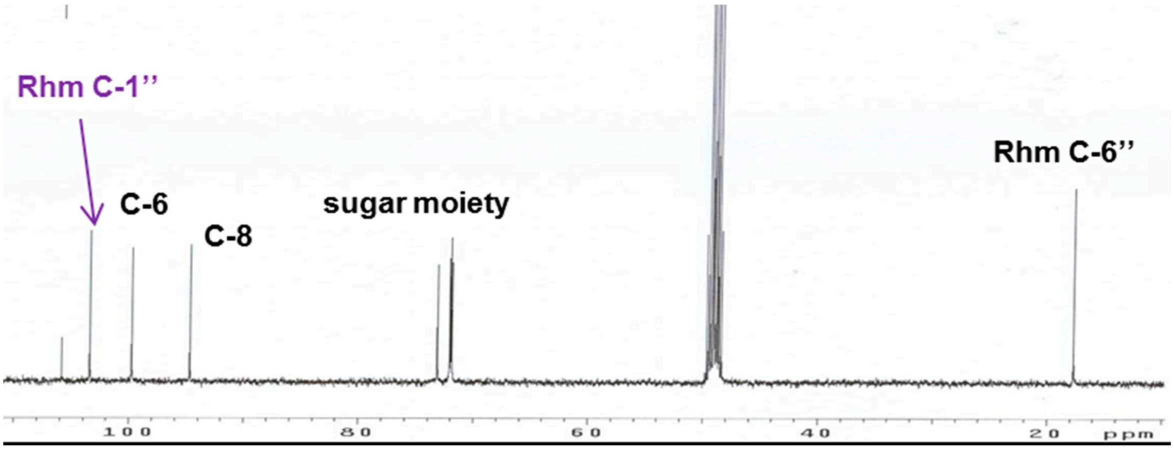

^{13}C-NMR specrrum-2

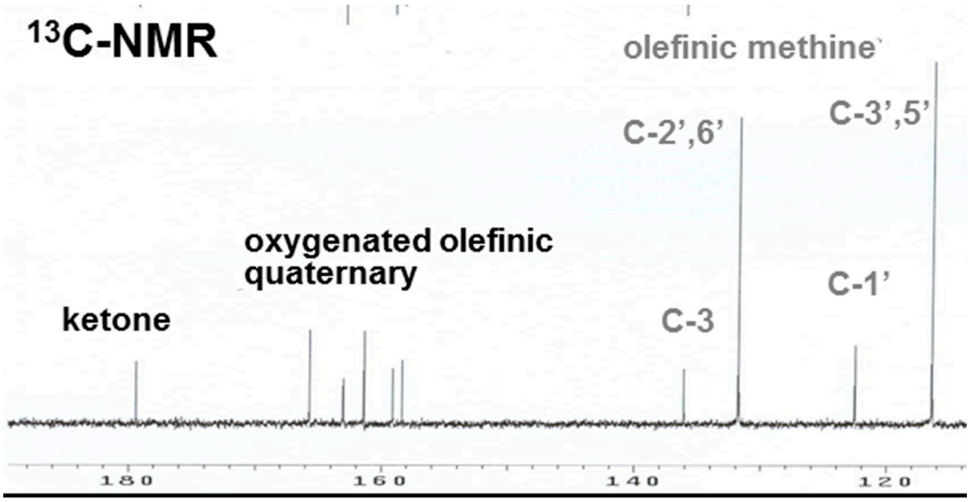

DEPT spectrum-1

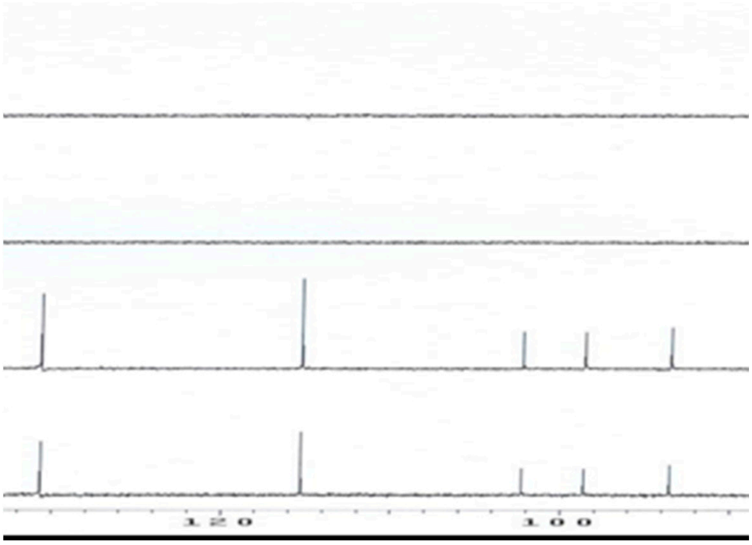

DEPT spectrum-2

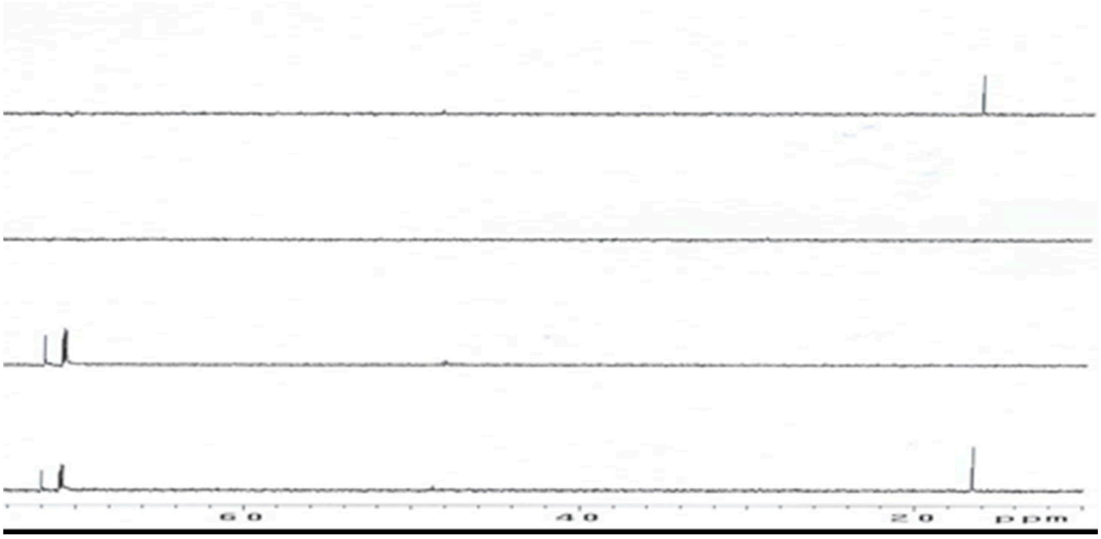

gHMBC spectrum

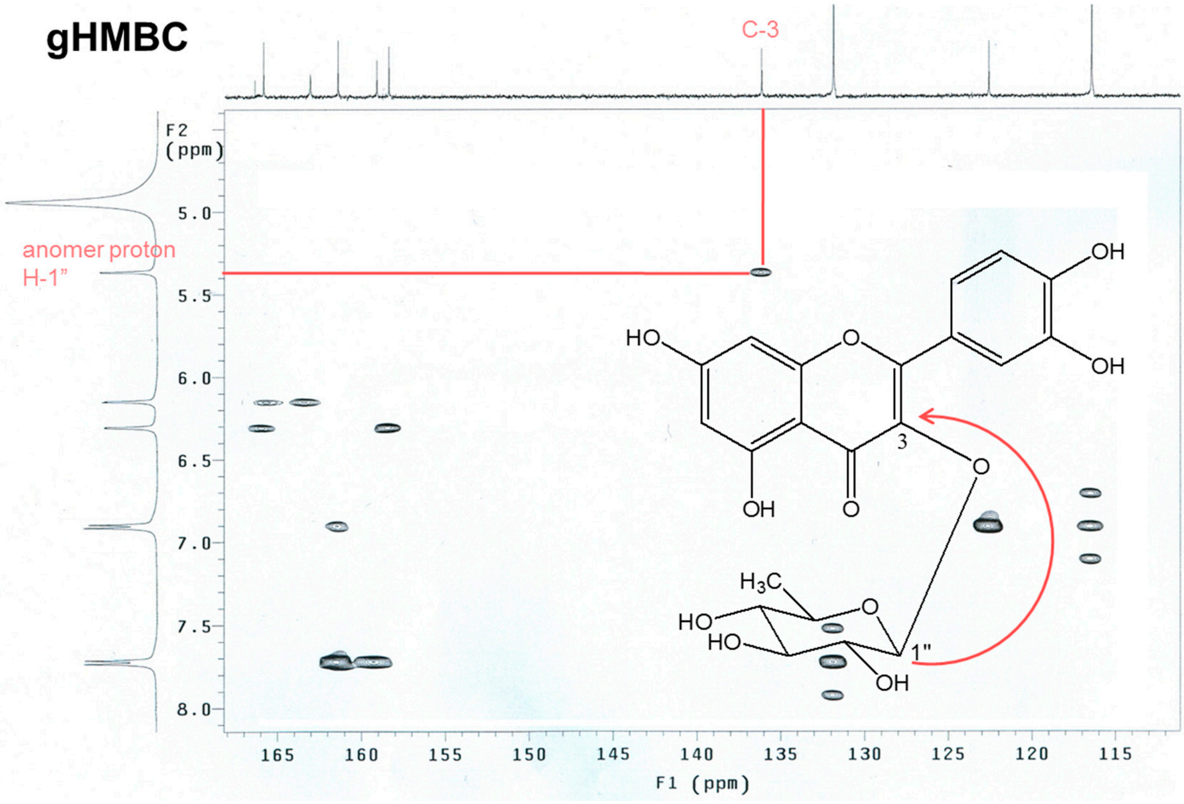

Compound **3 ((−)-epicatechin)**

^{1}H-NMR spectrum

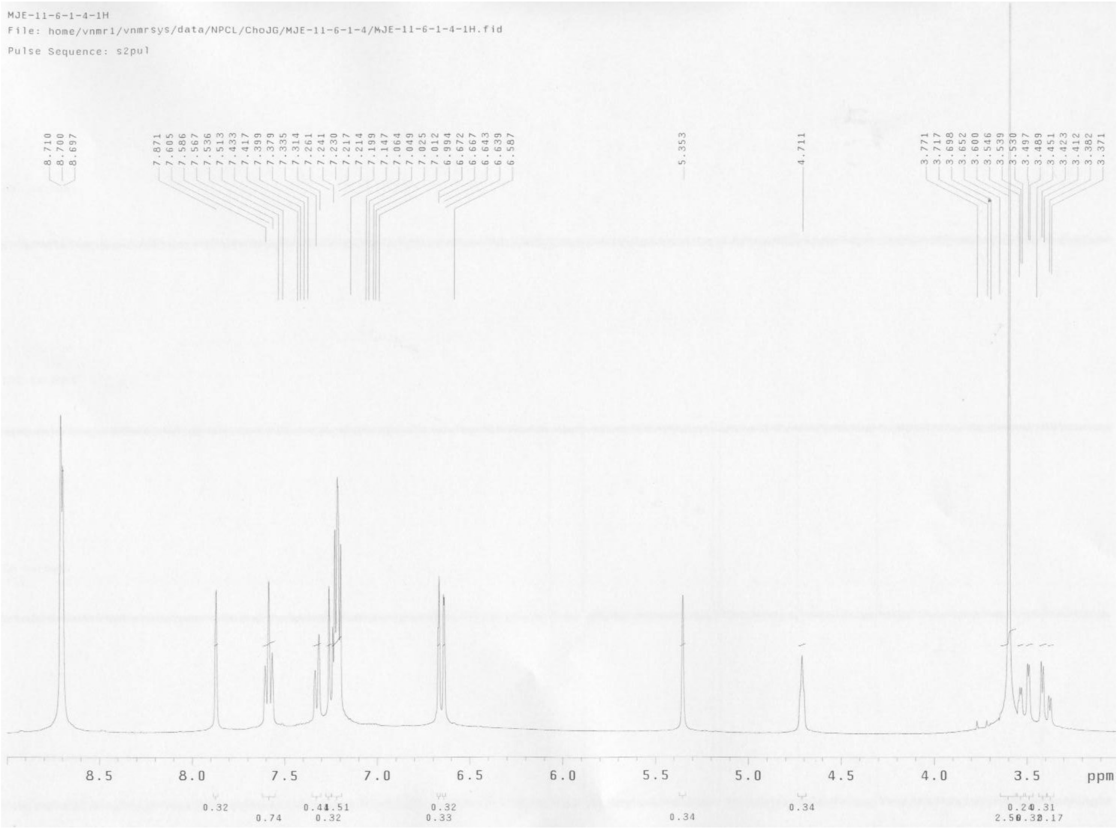

^{13}C-NMR spectrum

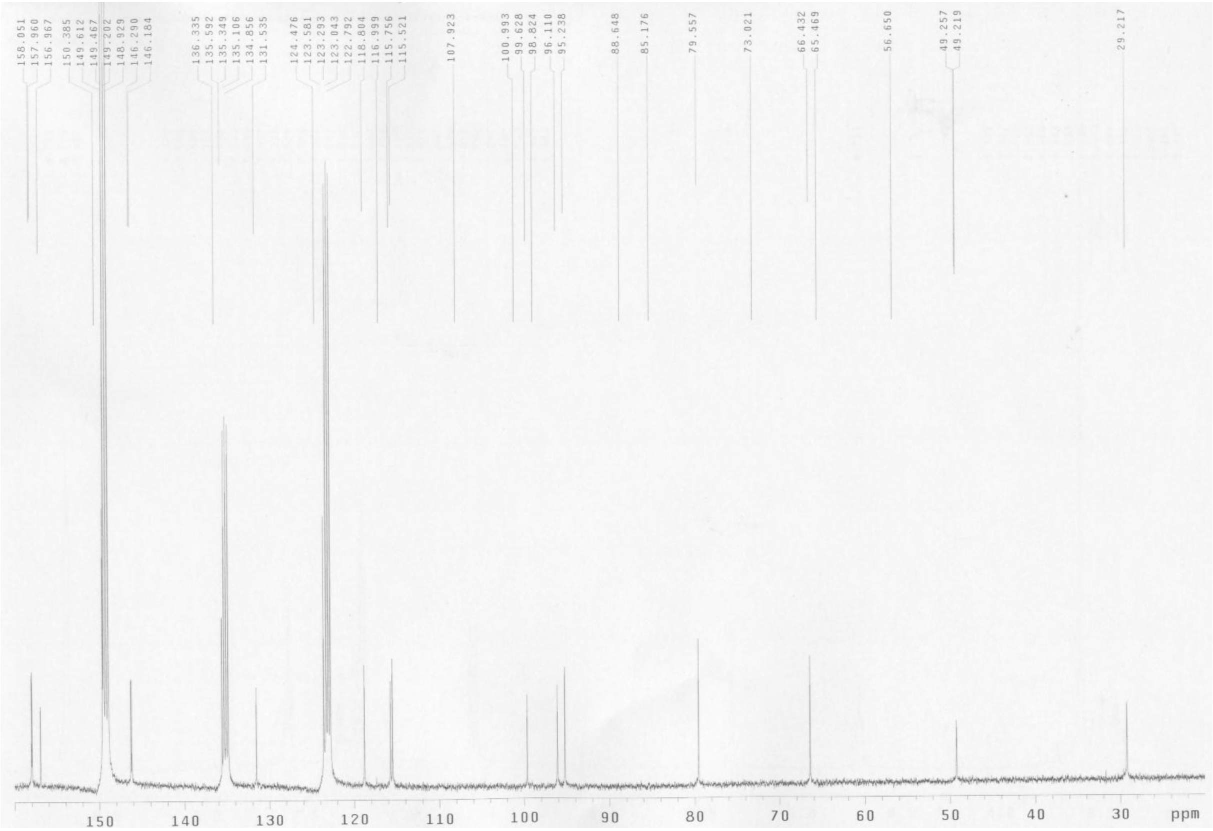

DEPT spectrum

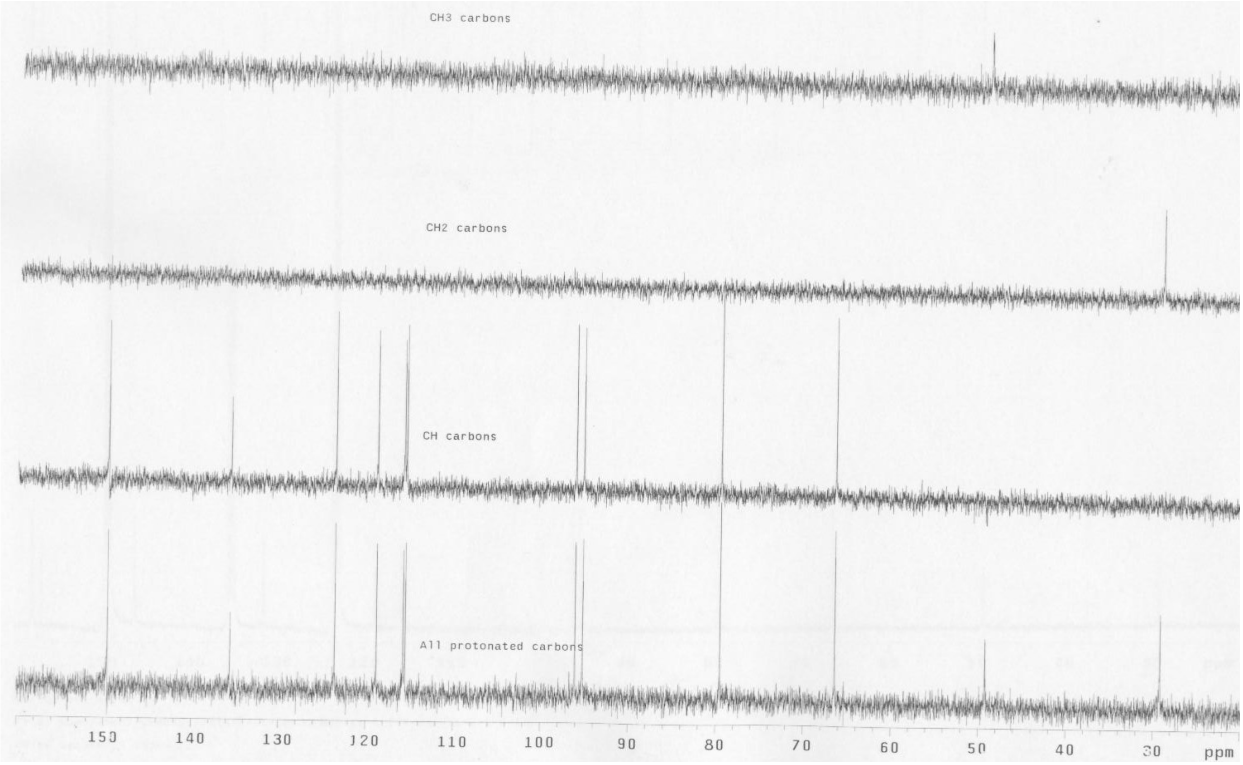

gHMBC spectrum

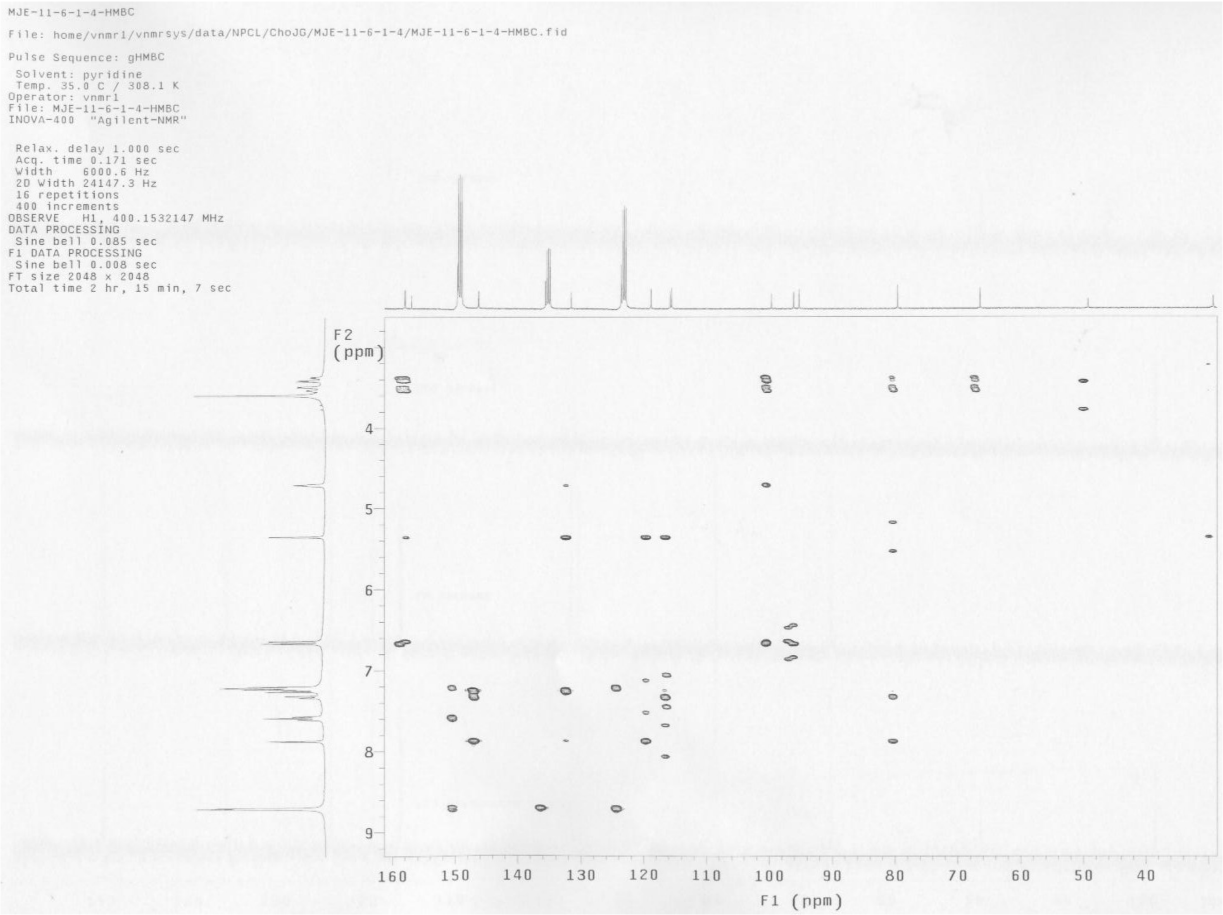

Compound **4 (5,3'-di-*O*-methyl-(−)-epicatechin)**

^{1}H-NMR spectrum

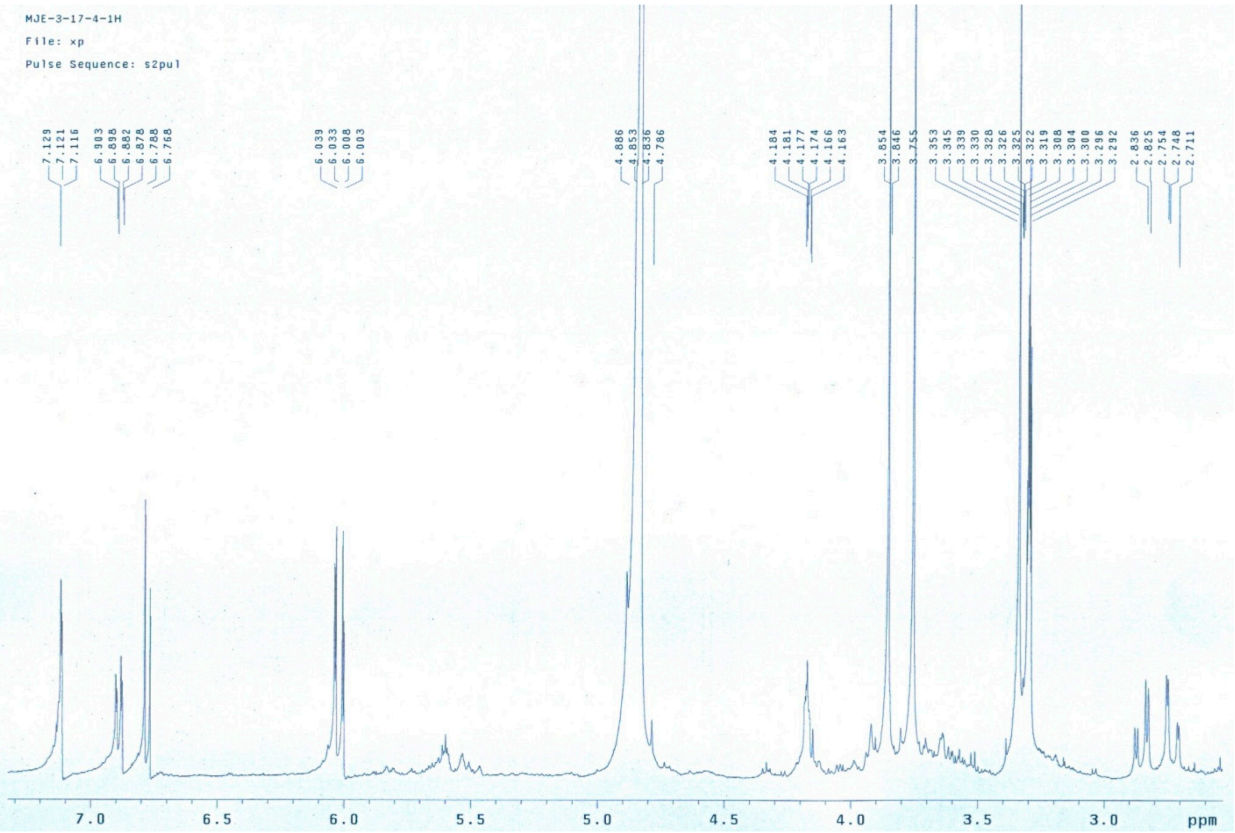

^{13}C-NMR spectrum

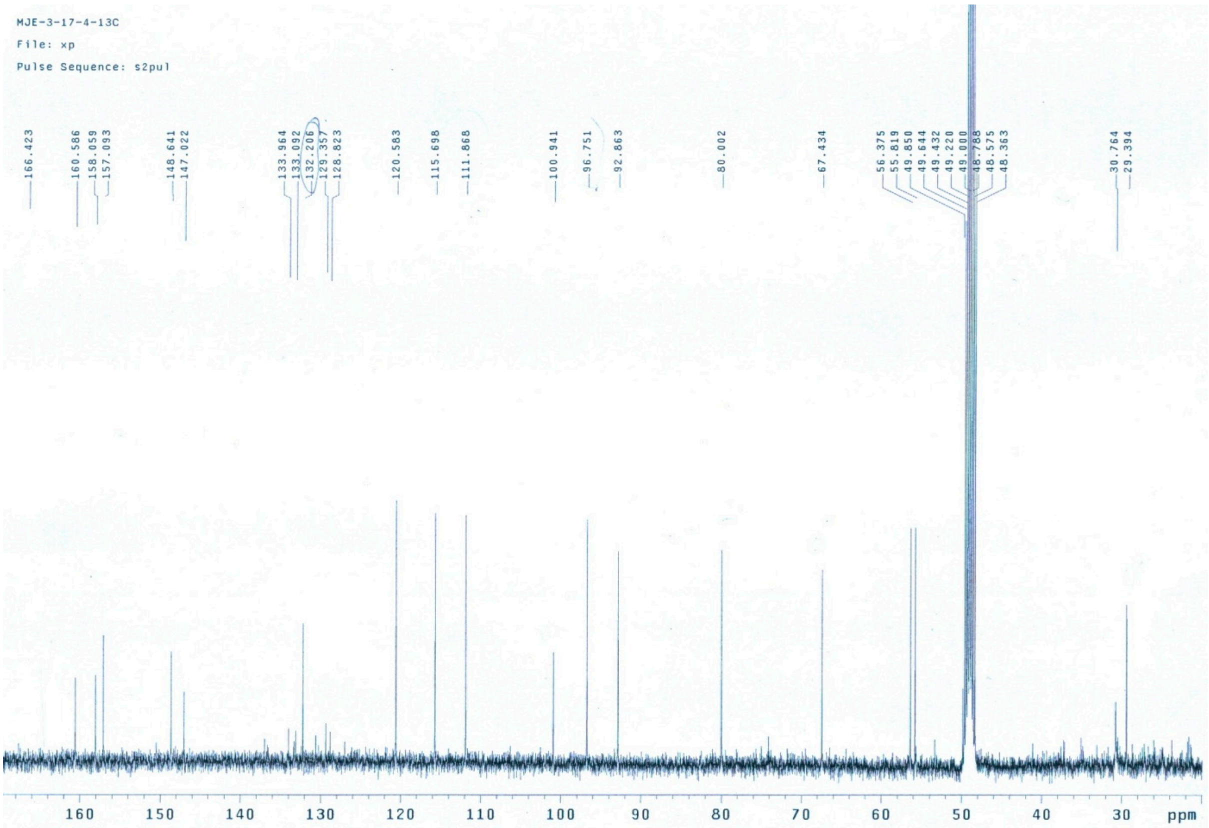

DEPT spectrum

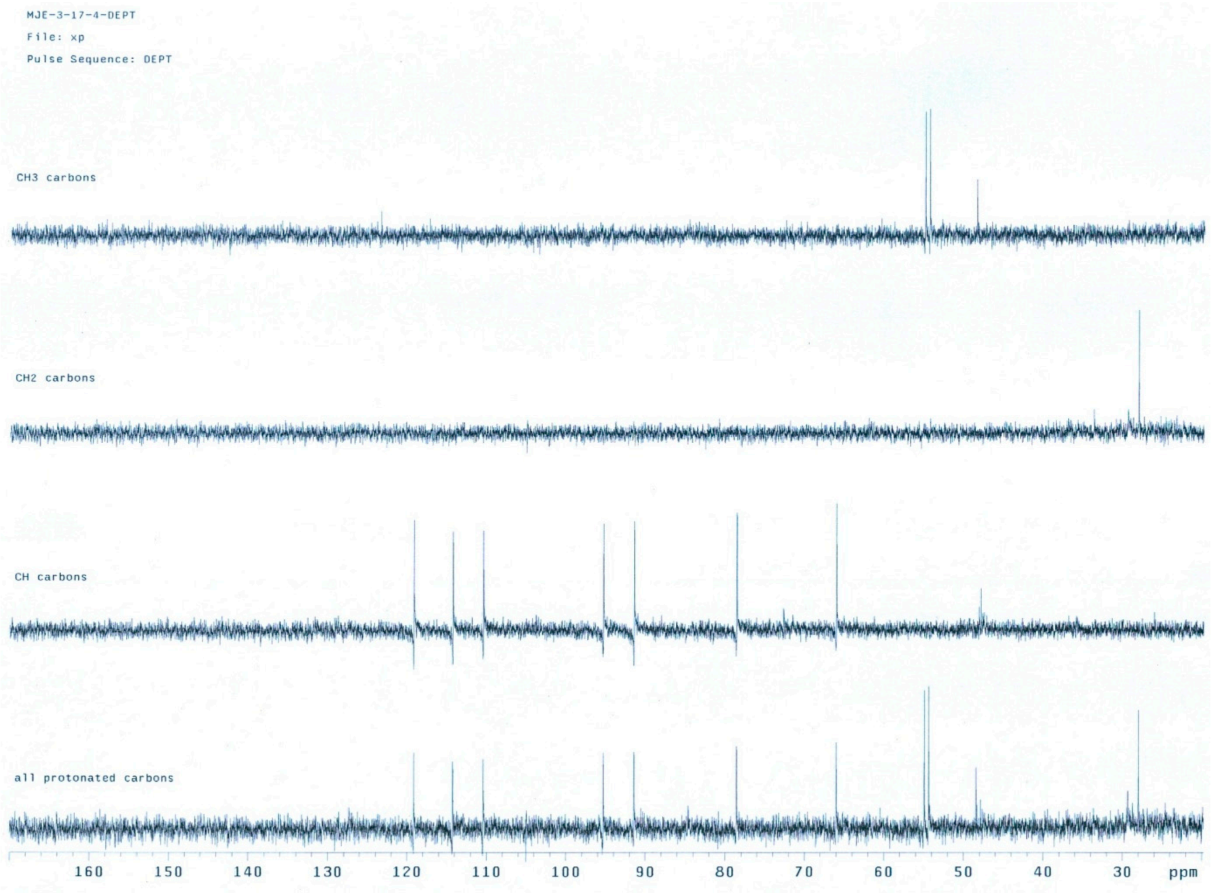

gHMBC spectrum

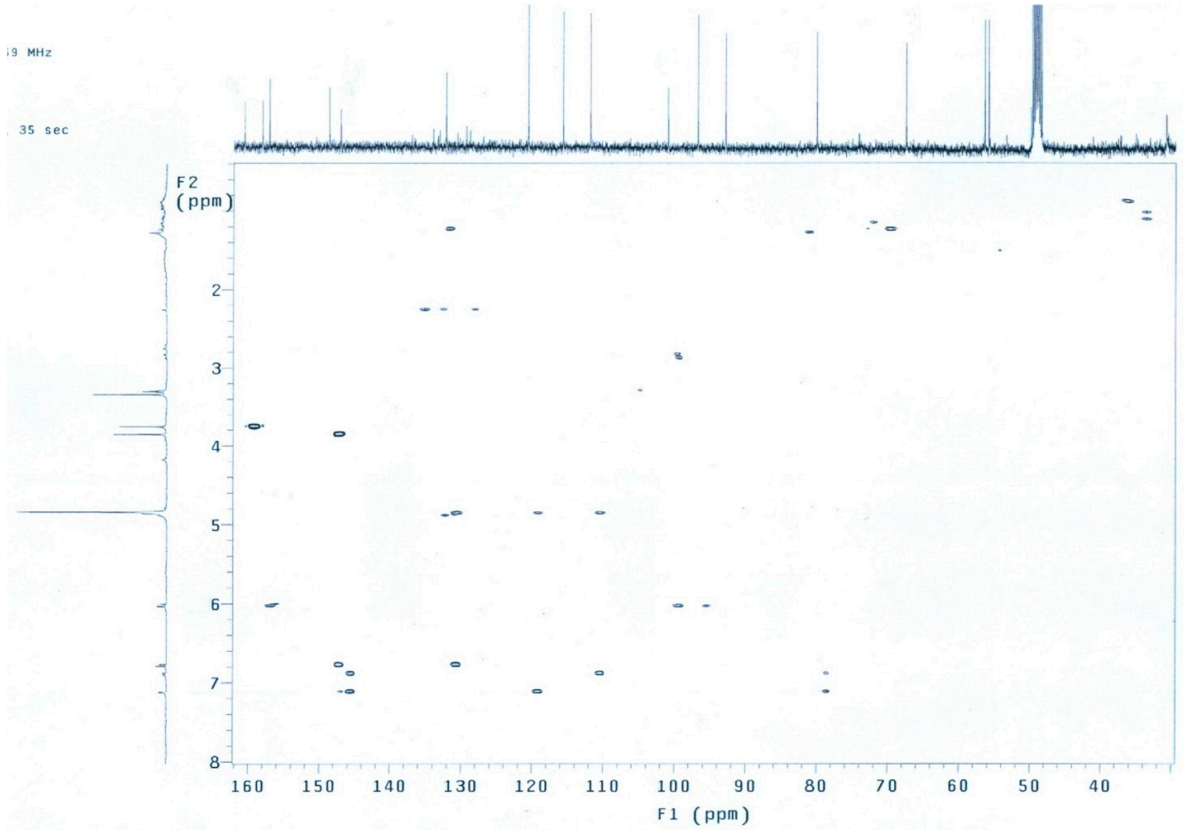

Compound **5 (5,7,3'-tri-*O*-methyl-(−)-epicatechin)**

^{1}H-NMR spectrum

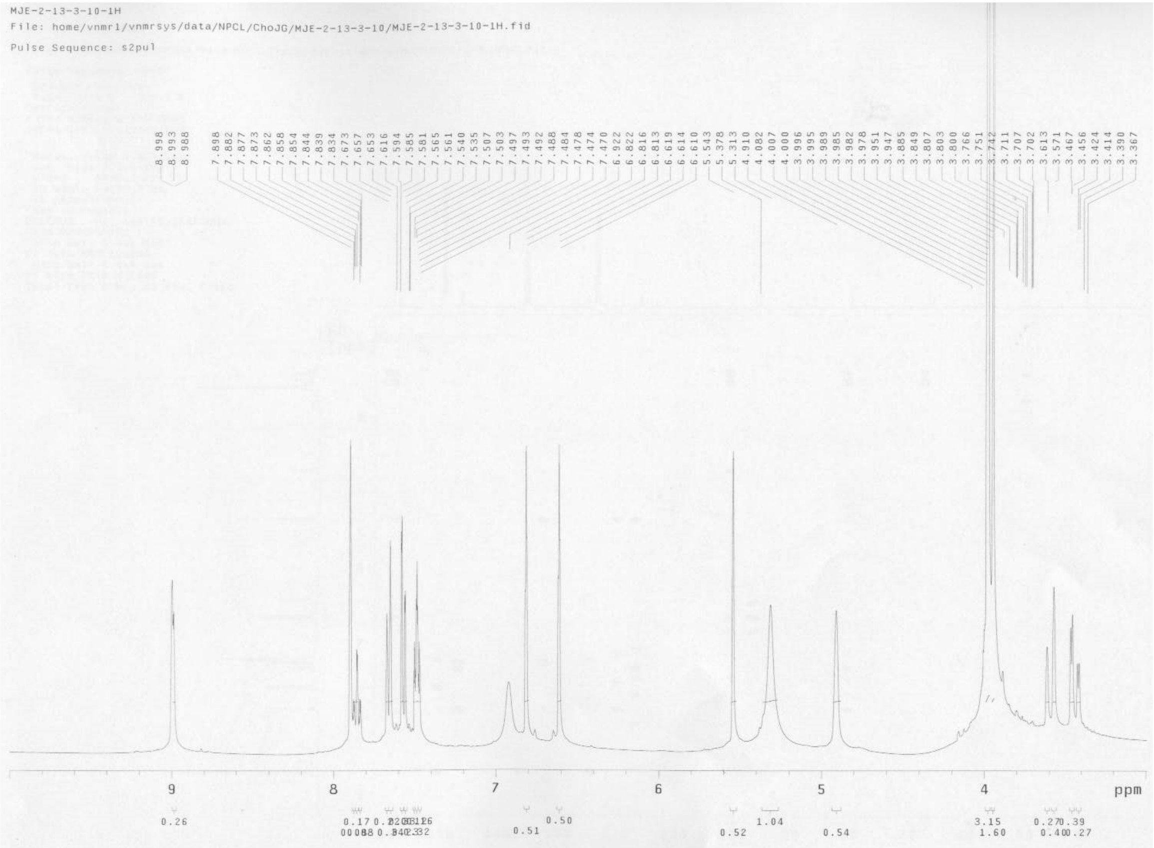

^{13}C-NMR spectrum

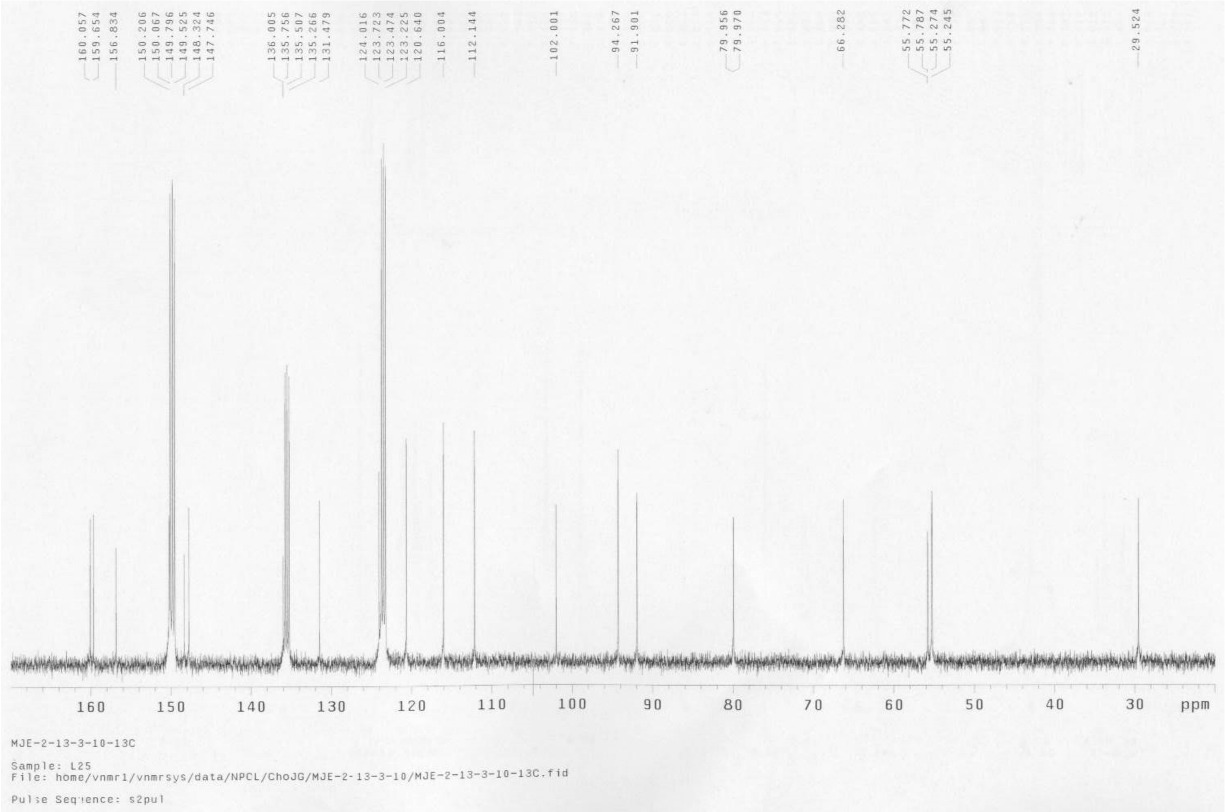

DEPT spectrum

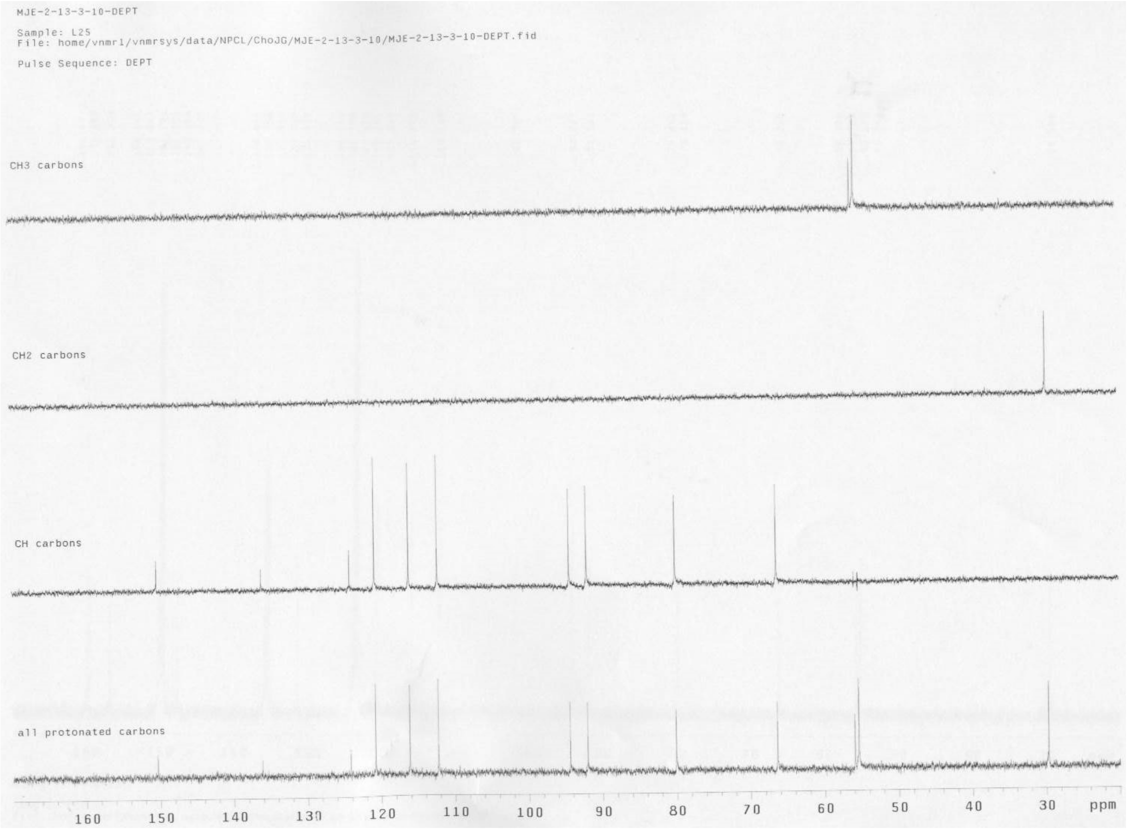

g-HMBC spectrum

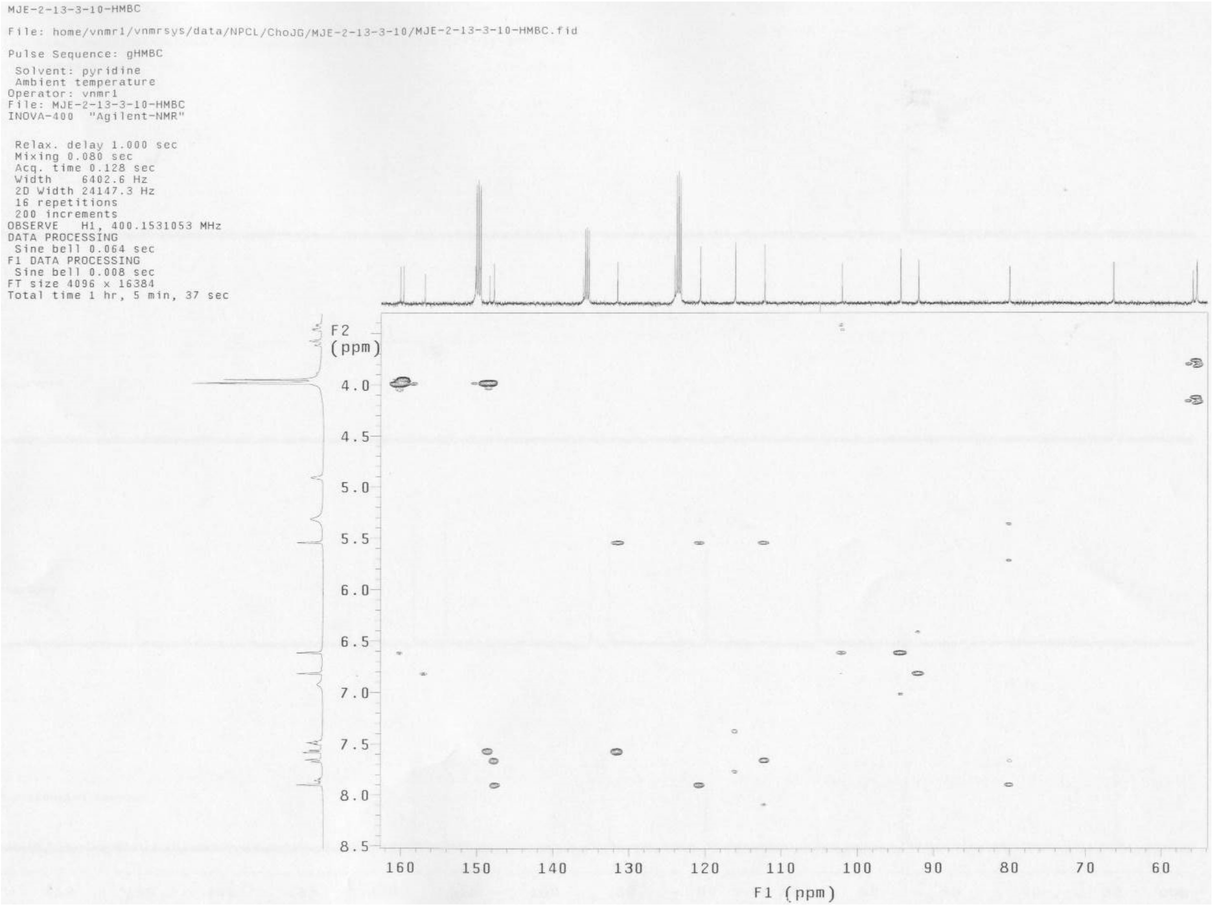

Compound **6 (5,7,-di-*O*-methyl-3',4'-methylenedioxyflavan-3-ol)**

^{1}H-NMR spectrum

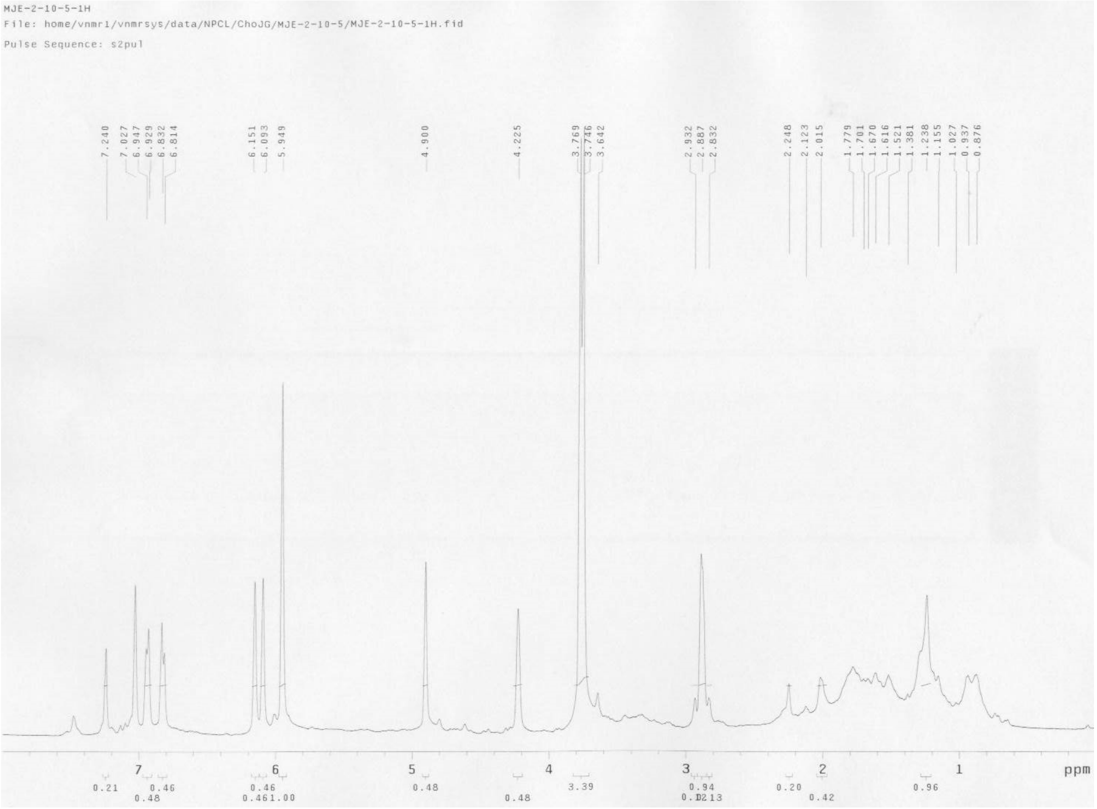

^{13}C-NMR spectrum

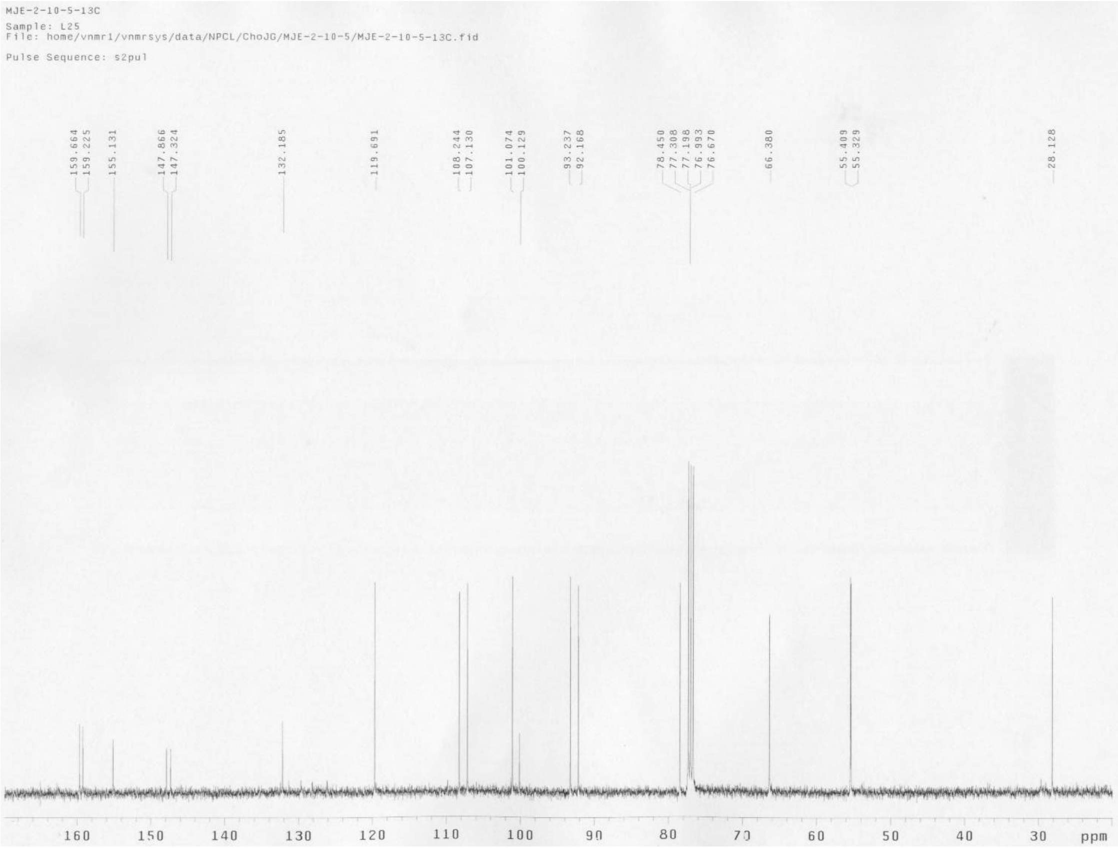

DEPT spectrum

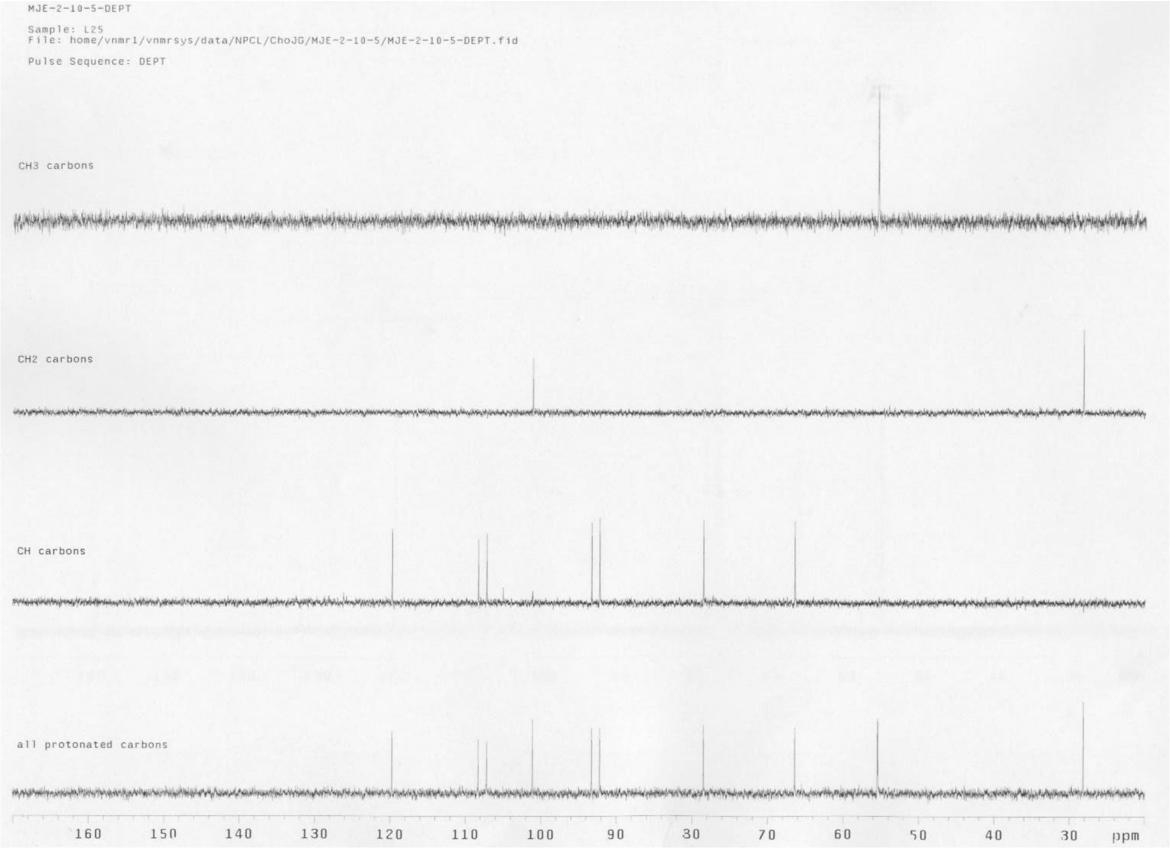

g-HMBC spectrum

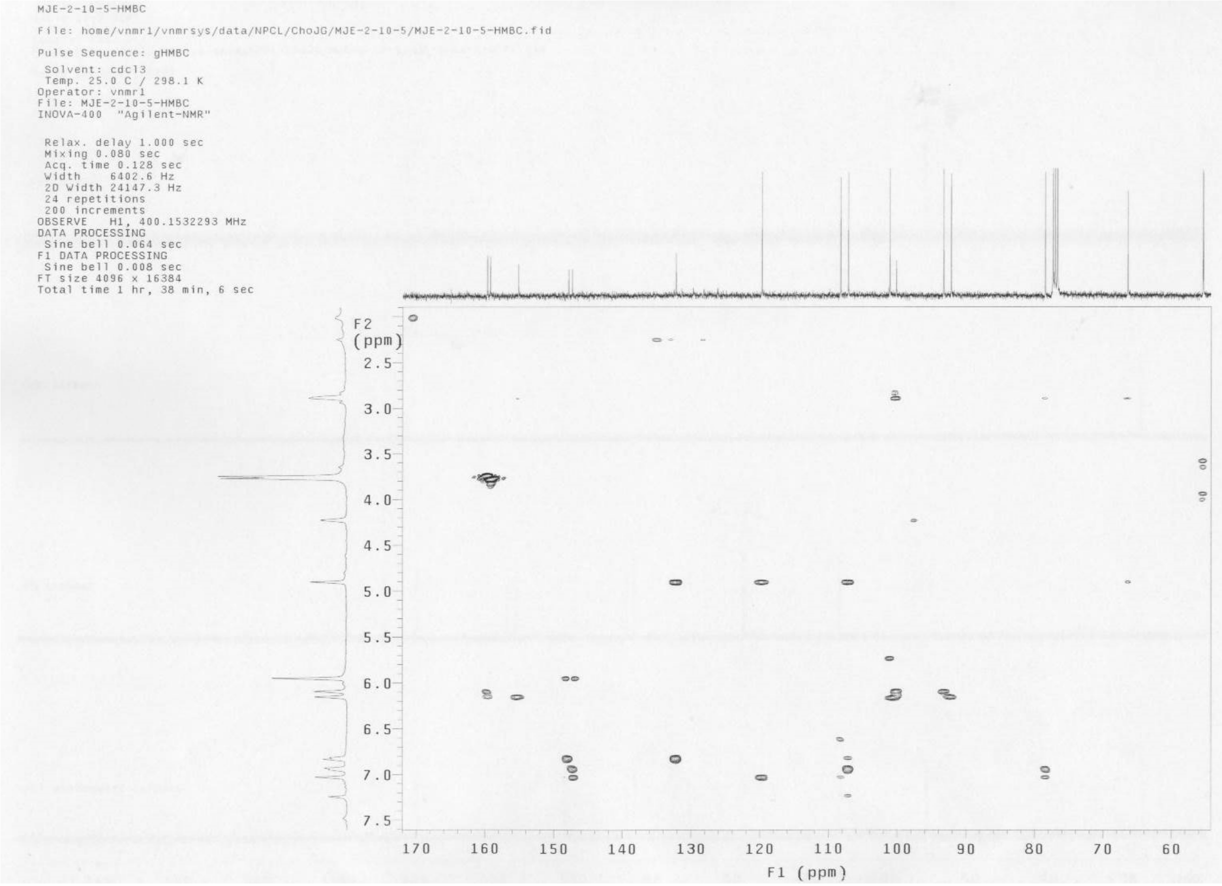

Supplement: Supplementary File 1 [file ijms-15-16418-s001.pdf]
